# Iron-Catalyzed C(sp^2^)–C(sp^3^) Cross-Coupling of Aryl Chlorobenzoates with Alkyl Grignard Reagents

**DOI:** 10.3390/molecules25010230

**Published:** 2020-01-06

**Authors:** Elwira Bisz, Michal Szostak

**Affiliations:** 1 Department of Chemistry, Opole University, 48 Oleska Street, 45-052 Opole, Poland; 2 Department of Chemistry, Rutgers University, 73 Warren Street, Newark, NJ 07102, USA

**Keywords:** iron, cross-coupling, aryl esters, C–O activation, Fe-catalysis, Kumada cross-coupling

## Abstract

Aryl benzoates are compounds of high importance in organic synthesis. Herein, we report the iron-catalyzed C(sp^2^)–C(sp^3^) Kumada cross-coupling of aryl chlorobenzoates with alkyl Grignard reagents. The method is characterized by the use of environmentally benign and sustainable iron salts for cross-coupling in the catalytic system, employing benign urea ligands in the place of reprotoxic NMP (NMP = N-methyl-2-pyrrolidone). It is notable that high selectivity for the cross-coupling is achieved in the presence of hydrolytically-labile and prone to nucleophilic addition phenolic ester C(acyl)–O bonds. The reaction provides access to alkyl-functionalized aryl benzoates. The examination of various O-coordinating ligands demonstrates the high activity of urea ligands in promoting the cross-coupling versus nucleophilic addition to the ester C(acyl)–O bond. The method showcases the functional group tolerance of iron-catalyzed Kumada cross-couplings.

## 1. Introduction

Iron catalyzed cross-couplings have recently emerged as an extremely valuable platform for organic synthesis [1,2,3,4,5,6,7,8,9,10,11,12,13,14,15,16,17,18,19]. Of particular interest is the high natural abundance of iron [11,12,13], which in combination with the low toxicity of iron salts and their facile removal from post-reaction mixtures makes it attractive for large-scale industrial processes [19]. The beneficial effect of iron for cross-coupling reactions extends far beyond its economical and sustainable ecological profile, and it is demonstrated by the establishment of the traditionally challenging C(sp^2^)–C(sp^3^) cross-couplings employing alkyl Grignard reagents possessing β-hydrogens that are not easily accomplished using other transition metals [14,15,16,17,18]. In this regard, the iron-NMP (NMP = N-methyl-2-pyrrolidone) system elegantly pioneered by Fürstner and co-workers represents by far the most viable option for iron cross-coupling chemistry [20,21,22,23,24,25,26,27,28,29,30,31,32,33]. The success of the iron-NMP reagent relies in large part on the outstanding functional group tolerance of this catalyst, the high toxicity of NMP notwithstanding [34,35]. In this vein, our laboratory has reported iron-catalyzed cross-couplings with alkyl Grignard reagents using benign urea ligands that represent an effective alternative to the reprotoxic NMP [36,37,38,39,40,41,42,43].

**Scheme 1:**
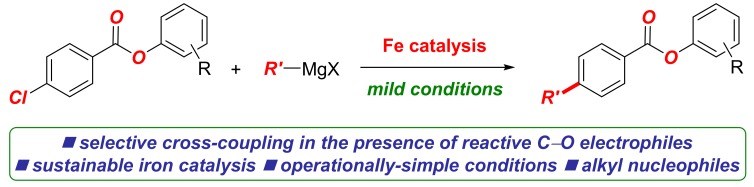
Iron-catalyzed C(sp^2^)–C(sp^3^) cross-coupling of aryl chlorobenzoates with alkyl Grignard reagents (this study).

**Scheme 2:**
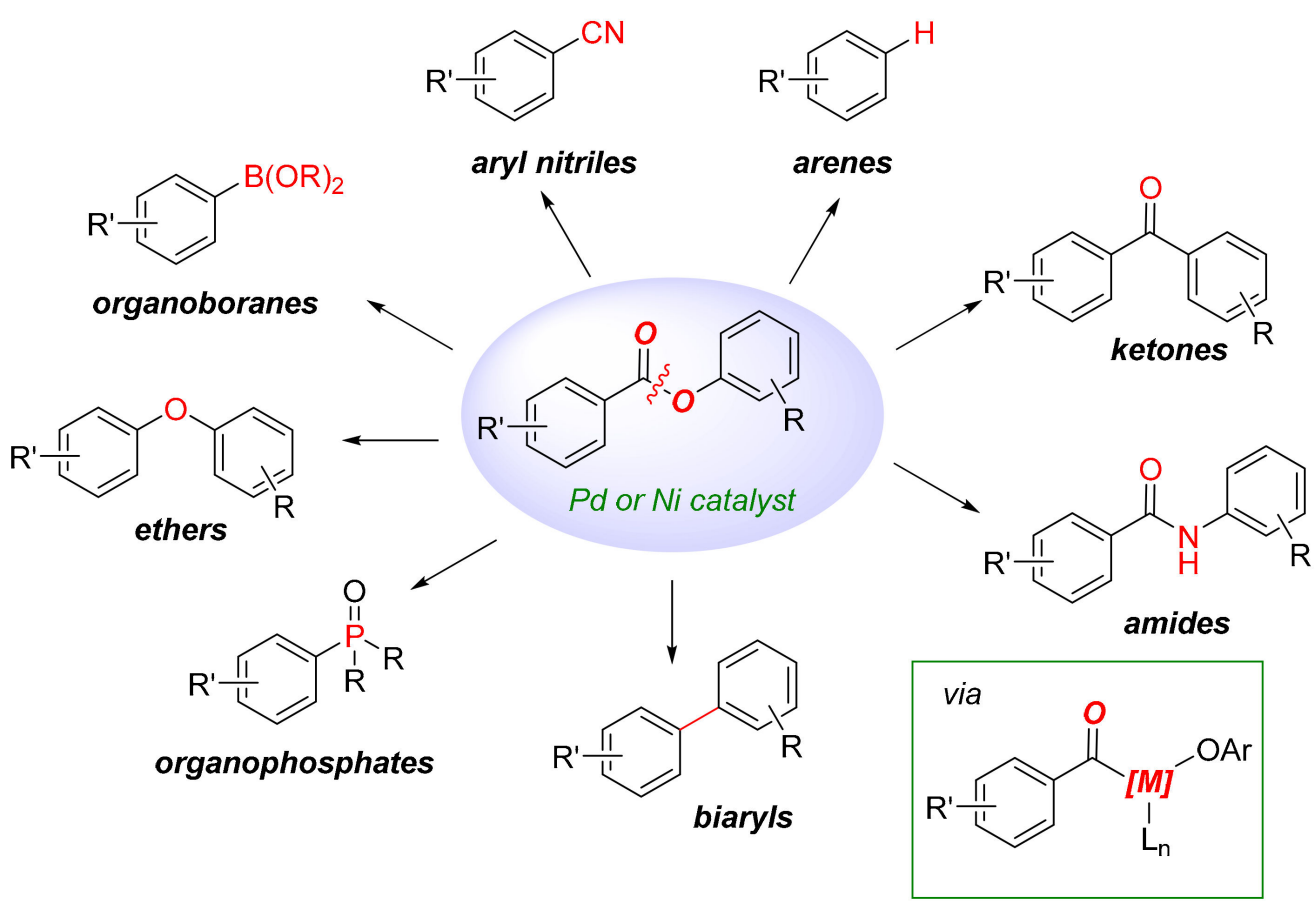
The important transformations via substituted aryl esters, the products of this study.

In this Special Issue on *Recent Advances in Iron Catalysis*, we detail our findings on the development of the iron-catalyzed cross-coupling of aryl chlorobenzoates with alkyl Grignard reagents (Scheme 1). The reaction is notable for several reasons: (1) the method allows for the synthesis of alkyl-functionalized aryl benzoates, which represent compounds of high importance in organic synthesis (Scheme 2); (2) the method demonstrates the exceptional functional group tolerance of the iron system, wherein the selective Kumada cross-coupling is achieved in the presence of the hydrolytically labile and prone to nucleophilic addition C(acyl)–O ester moiety. This model system is well suited for the examination of various O-coordinating ligands in promoting the cross-coupling versus nucleophilic addition to the ester bond. More broadly, the reaction showcases the functional group tolerance in the industrially important iron-catalyzed Kumada cross-couplings.

## 2. Results

We became interested in developing the iron-catalyzed cross-coupling of aryl chlorobenzoates as part of our program in iron catalysis [36,37,38,39,40,41,42,43] and the cross-coupling of C(acyl)–X (X = N, O) electrophiles [44,45]. Recently, several groups have reported methods for the nickel and palladium-catalyzed C(acyl)–O bond activation of aryl benzoates, leading to the selective formation of acyl-metal intermediates (Scheme 2, box) [46,47,48,49,50,51,52,53,54,55,56,57,58,59,60,61,62,63,64]. While aryl benzoates have long been established as electrophiles in nucleophilic addition to the ester bond via tetrahedral intermediates owing to the increased electrophilicity of the ester bond due to O_lp_ to Ar conjugation [65], the recent advances in accessing acyl metals from aryl benzoates significantly expand the utility of this class of carboxylic acid derivatives in organic synthesis. Thus, the direct iron-catalyzed Kumada cross-coupling would provide an attractive method for the functionalization of the aromatic ring; however, perhaps not surprisingly given the high reactivity of the C(acyl)–O bond, generally applicable methods for the C(sp^2^)–C(sp^3^) Kumada cross-coupling of aryl benzoates have been elusive.

At the outset, we probed the model reaction between phenyl 4-chlorobenzoate (**1a**) and ethylmagnesium chloride in the presence of benign DMI (DMI = 1,3-dimethyl-2-imidazolidinone) (Table 1). Under standard conditions, the cross-coupling proceeded in 27% yield with the remaining mass balance corresponding to the alcohol product (Table 1, entry 1). Lowering the equivalents of the Grignard reagent had no impact on the reaction efficiency (Table 1, entry 2). After experimentation, we found that the slow addition of the close to equimolar quantity of the Grignard reagent afforded the desired cross-coupling product in 65% yield (Table 1, entry 3). Interestingly, using an excess of DMI led to lower cross-coupling efficiency, which was likely due to facilitating the nucleophilic addition to the carbonyl group (Table 1, entry 4). DMI improves the coupling efficiency; however, this additive is not required, as demonstrated by the cross-coupling in its absence (Table 1, entries 3–6). Furthermore, using Grignard as the limiting reagent as well as extending the addition time had a deleterious effect on the cross-coupling (Table 1, entries 7–8). Likewise, increasing the iron loading to generate the active organoferrate in excess gave no observable increase in the reaction efficiency (Table 1, entries 9–10). Further, we note that an efficient reaction ensues at −40 °C (Table 1, entry 11), while negligible conversion was observed at −78 °C (Table 1, entry 12). Importantly, control reactions in the absence of iron, with and without DMI (Table 1, entries 13–14), resulted in no cross-coupling with the alcohol formed as the sole reaction product, thereby highlighting the key role of iron to promote the cross-coupling. Finally, for comparison purposes, we tested NMP as the additive (Table 1, entry 15). Interestingly, NMP resulted in lower cross-coupling efficiency than DMI (vide infra), highlighting the beneficial effect of this ligand beyond its favorable toxicological profile (cf. NMP). 

Then, we examined the scope of the optimized iron catalytic system as outlined in Table 2. We were pleased to find that neutral as well as electron-rich aryl 4-chlorobenzoates, such as 4-*tert*-butyl and 4-methoxy, enabled the chemoselective cross-coupling in good yields (Table 2, entries 1–3). Furthermore, electron-deficient aryl 4-chlorobenzoates, such as 4-fluoro, are also tolerated, albeit the cross-coupling product is obtained in lower yield (Table 2, entry 4). As expected, the reactivity trend mirrors the electronic properties of the aryl ester in that electron-deficient aryl substituents increase O_lp_ to Ar conjugation, leading to the lower yield in the cross-coupling. Pleasingly, we found that both sterically-hindered 2-methyl and 2,6-dimethyl aryl 4-chlorobenzoates are well-tolerated (Table 2, entries 5–6) and result in significantly improved yields for the cross-coupling as a result of steric shielding of the C(acyl)–O bond. Thus, we recommend that electron-rich or sterically hindered aryl benzoates are used for the cross-coupling to minimize the formation of the alcohol side products. 4-Chlorophenyl 4-chlorobenzoate is not a suitable substrate due to nucleophilic addition. The scope of Grignard reagents was also briefly examined (Table 2, entries 7–10). As such, longer primary alkyl Grignard reagents such as hexyl or tetradecyl gave the cross-coupling products in high yields (Table 2, entry 7–8). The cross-coupling of more sterically hindered secondary Grignard reagents is feasible; however, it leads to modest yield (Table 2, entry 9). Finally, we were pleased to find that the challenging phenethyl Grignard reagent that is prone to β-hydride elimination is also a competent nucleophile for this cross-coupling protocol (Table 2, entry 10), attesting to the efficiency of the cross-coupling. At present, cross-coupling of 3-substituted aryl chlorobenzoates is not feasible due to facile hydrolysis.

**Scheme 3:**
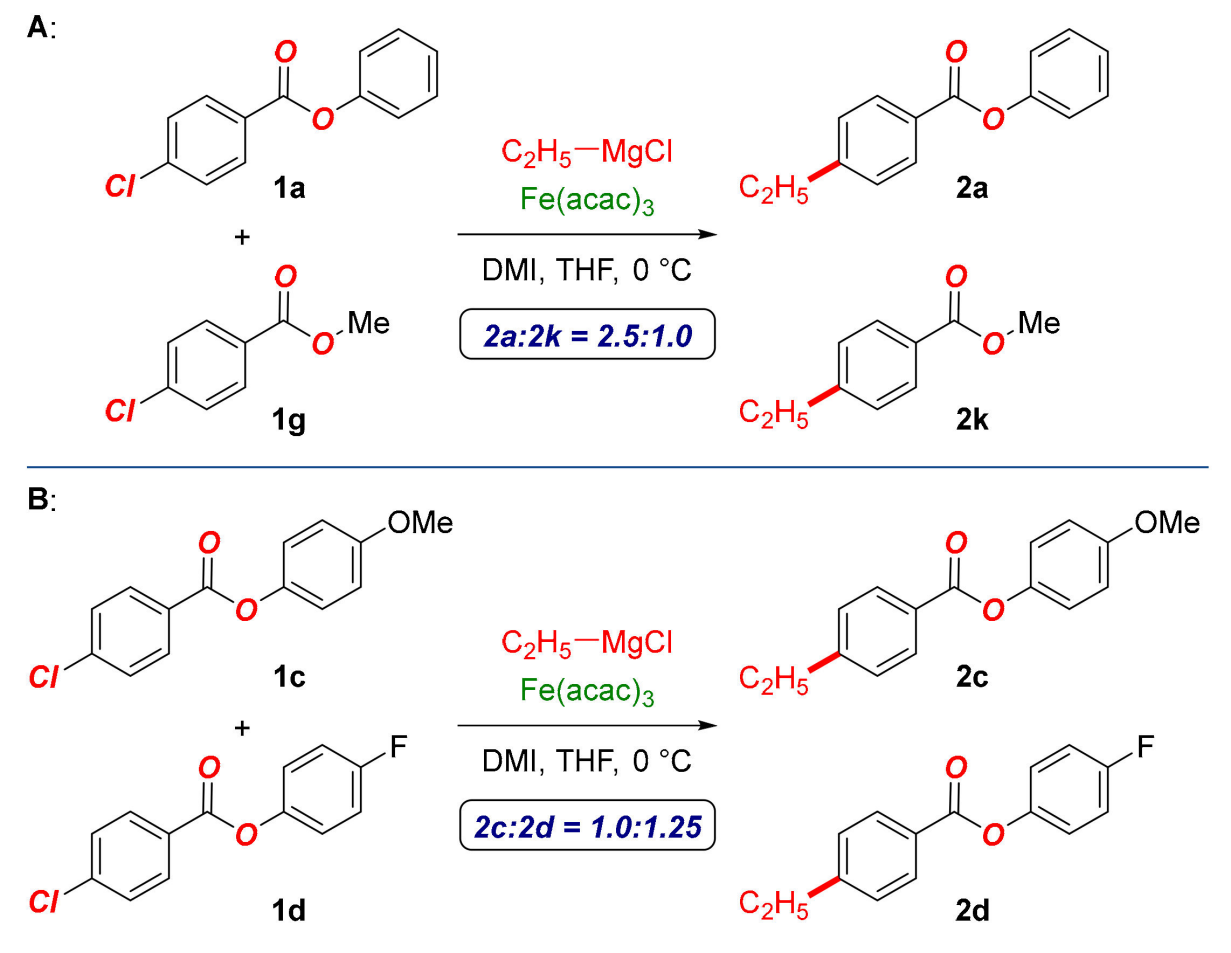
Competition experiments. (**A**) Competition experiments between phenyl and methyl ester (OPh:OMe = 2.5:1.0) revealed that aryl esters are more reactive than their alkyl counterparts, which is consistent with the facility of oxidative addition. (**B**) Similarly, competition between electron-rich and electron-deficient aryl esters (4-MeO:4-F = 1.0:1.25) revealed that electron-deficient arenes are more reactive

Next, intermolecular competition studies were performed to gain insight into the selectivity of this cross-coupling (Scheme 3). (A) Competition experiments between phenyl and methyl ester (OPh:OMe = 2.5:1.0) revealed that aryl esters are more reactive than their alkyl counterparts, which is consistent with the facility of oxidative addition. (B) Similarly, competition between electron-rich and electron-deficient aryl esters (4-MeO:4-F = 1.0:1.25) revealed that electron-deficient arenes are more reactive. This observation is in agreement with the O-aryl ester activating the aromatic ring for the cross-coupling; however, its increased electrophilicity leads to a competing nucleophilic addition to give the alcohol products. The formation of the alcohol could be minimized by using sterically hindered or electron-rich aromatic esters. 

**Figure 1:**
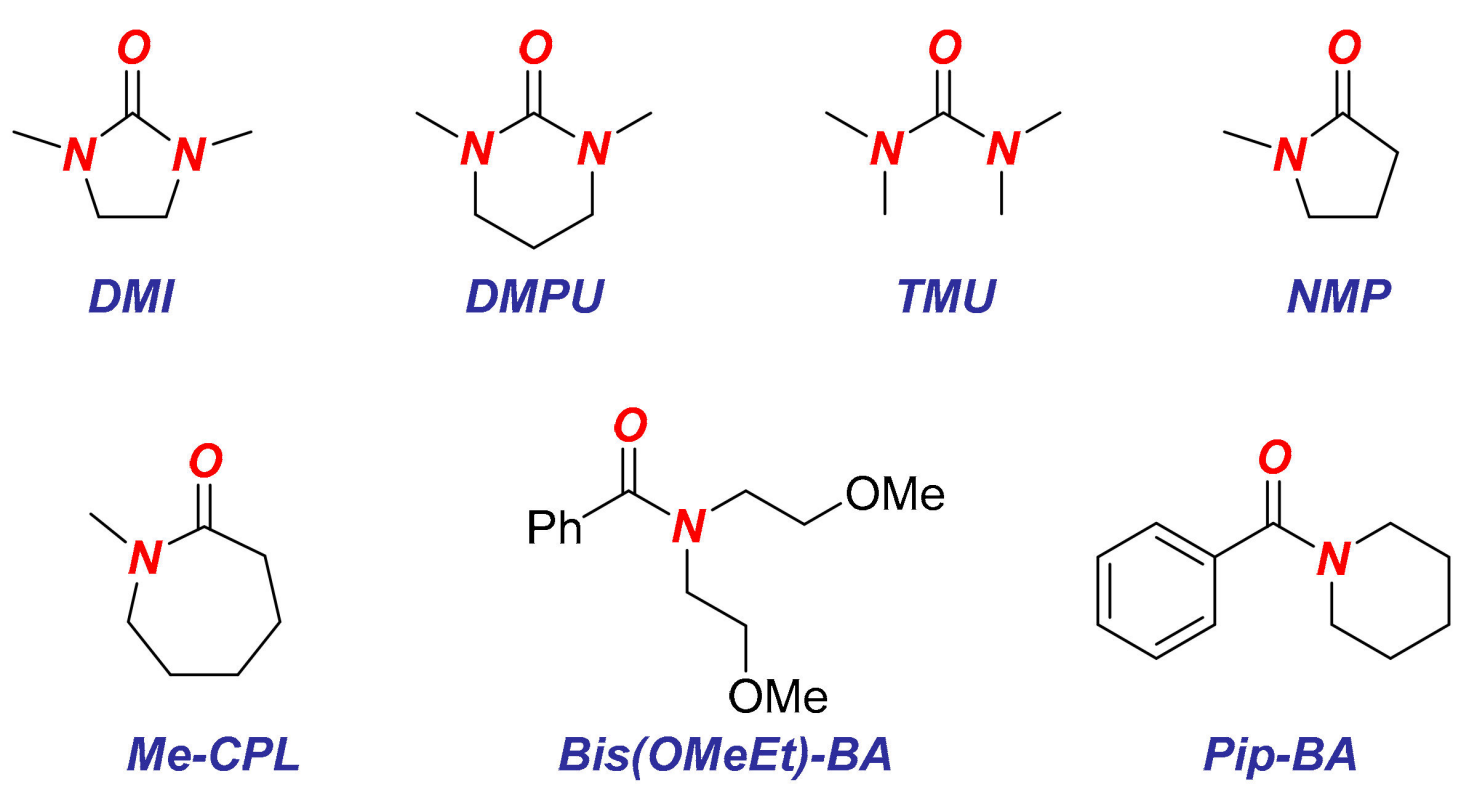
Structures of ligands used.

Finally, we have probed the effect of various additives on the cross-coupling (Table 3 and Figure 1). At present, one of the major challenges in iron-catalyzed C(sp^2^)–C(sp^3^) cross-coupling is replacing the reprotoxic NMP by benign yet effective additives. The present system compares the cross-coupling efficiency versus nucleophilic addition, thereby indirectly measuring the ligand effect. Our study demonstrates that urea ligands such as DMI, DMPU (DMPU = 1,3-dimethyl-3,4,5,6-tetrahydro-2(1*H*)-pyrimidinone) and TMU (TMU = 1,1,3,3-tetramethylurea) are more reactive than NMP in the cross-coupling (Table 3, entries 1–4), while *N*-methylcaprolactam shows comparable reactivity to NMP (Table 3, entry 5). In contrast, the recently reported by our group *N*,*N*-bis(2-methoxyethyl)benzamide (Table 3, entry 6) and phenyl(piperidin-1-yl)methanone (Table 3, entry 7) appear to be less reactive than NMP [10]; however, ester hydrolysis is not observed in these cases, which may lead to unusual selectivity in the cross-coupling. 

## 3. Discussion

In summary, we have reported the iron-catalyzed C(sp^2^)–C(sp^3^) Kumada cross-coupling of aryl chlorobenzoates with alkyl Grignard reagents. The iron-catalyzed cross-coupling reactions have gained significant momentum due to the beneficial environmental and sustainability profile compared to precious metals. However, what is equally important is the fact that iron catalysis enables cross-coupling reactions that are difficult or impossible to achieve with other metals, the prime example being the industrially-relevant C(sp^2^)–C(sp^3^) Kumada cross-coupling. The present study expands the scope of benign iron-catalyzed cross-couplings with urea ligands as replacements for toxic NMP to embrace the functional group tolerance of highly reactive aryl benzoates without cleavage of the sensitive C(acyl)–O bond. Future studies will be focused on expanding the scope of iron-catalyzed cross-couplings and the design of new amide-based ligands for iron catalysis.

## 4. Materials and Methods

### 4.1. General Information 

All compounds reported in the manuscript are commercially available or have been previously described in the literature unless indicated otherwise. All experiments involving iron were performed using standard Schlenk techniques under argon or nitrogen atmosphere unless stated otherwise. All esters have been prepared by standard methods [66]. All yields refer to yields determined by ^1^H-NMR and/or GC/MS using an internal standard (optimization) and isolated yields (preparative runs) unless stated otherwise. ^1^H-NMR and ^13^C-NMR data are given for all compounds in the Experimental section for characterization purposes. ^1^H-NMR, ^13^C-NMR, and HRMS data are reported for all new compounds. All products have been previously reported, unless stated otherwise. Spectroscopic data matched literature values. General methods have been published [36,37,38,39,40,41,42,43]. All new compounds have been characterized by established guidelines by ^1^H-NMR, ^13^C-NMR, HRMS, and Mp as appropriate.

### 4.2. General Procedure for Iron-Catalyzed C(sp^2^)–C(sp^3^) Cross-Coupling

An oven-dried vial equipped with a stir bar was charged with an ester substrate (neat, typically, 0.50 mmol, 1.0 equiv) and Fe(acac)_3_ (typically, 5 mol%), which was placed under a positive pressure of argon and subjected to three evacuation/backfilling cycles under vacuum. Tetrahydrofuran (0.15 M) and ligand were sequentially added with vigorous stirring at room temperature, the reaction mixture was cooled to 0 °C, a solution of Grignard reagent (typically, 1.05 equiv) was added dropwise over 60 min with vigorous stirring, and the reaction mixture was stirred for the indicated time at 0 °C. After the indicated time, the reaction mixture was diluted with HCl (1.0 *N*, 1.0 mL) and Et_2_O (1 × 30 mL), and the organic layer was extracted with HCl (1.0 *N*, 2 × 10 mL), dried, and concentrated. The sample was analyzed by ^1^H-NMR (CDCl_3_, 400 MHz) and GC-MS to obtain the conversion, yield and, selectivity using an internal standard and comparison with authentic samples. Purification by chromatography on silica gel afforded the title product.

### 4.3. General Procedure for Determination of Relative Reactivity

According to the general procedure, an oven-dried vial equipped with a stir bar was charged with two chloride substrates (each 0.50 mmol, 1.0 equiv) and Fe(acac)_3_ (5 mol%), which was placed under a positive pressure of argon and subjected to three evacuation/backfilling cycles under vacuum. Tetrahydrofuran (0.15 M) and DMI (neat, 200 mol%) were sequentially added with vigorous stirring at room temperature, the reaction mixture was cooled to 0 °C, a solution of C_2_H_5_MgCl (2.0 M in THF, 0.25 mmol, 0.50 equiv) was added dropwise over 60 min with vigorous stirring, and the reaction mixture was stirred for 180 min at 0 °C. Following the standard work up, the sample was analyzed by ^1^H-NMR (CDCl_3_, 400 MHz) and GC-MS to obtain the conversion, yield, and selectivity using an internal standard and comparison with authentic samples.

### 4.4. Characterization Data for Starting Materials

*Phenyl 4-chlorobenzoate* (**1a**) [67]. Yield 95% (2.20 g). White solid. ^1^H-NMR (400 MHz, CDCl_3_) δ 8.13 (d, *J* = 8.8 Hz, 2H), 7.47 (d, *J* = 8.8 Hz, 2H), 7.45–7.39 (m, 2H), 7.30–7.25 (m, 1H), and 7.22–7.18 (m, 2H). ^13^C{1H} NMR (100 MHz, CDCl_3_) δ 164.47, 150.94, 140.26, 131.69, 129.69, 129.10, 128.19, 126.19, and 121.77.

*4-(Tert-Butyl)phenyl 4-chlorobenzoate* (**1b**). *New compound.* Yield 98% (2.84 g). White solid. Mp = 114–116 °C. ^1^H-NMR (400 MHz, CDCl_3_) δ 8.13 (d, *J* = 8.7 Hz, 2H), 7.46 (d, *J* = 8.6 Hz, 2H), 7.43 (d, *J* = 8.8 Hz, 2H), 7.12 (d, *J* = 8.8 Hz, 2H), and 1.34 (s, 9H). ^13^C{1H} NMR (100 MHz, CDCl_3_) δ 164.63, 149.01, 148.55, 140.16, 131.68, 129.06, 128.30, 126.59, 121.05, 34.67, and 31.58. HRMS (ESI/Q-TOF) *m/z*: [M + Na]^+^ calcd for C_17_H_17_ClO_2_Na 311.0815 found 311.0822. 

*4-Methoxyphenyl 4-chlorobenzoate* (**1c**) [68]. Yield 95% (2.50 g). White solid. ^1^H-NMR (400 MHz, CDCl_3_) δ 8.12 (d, *J* = 8.6 Hz, 2H), 7.47 (d, *J* = 8.6 Hz, 2H), 7.12 (d, *J* = 9.0 Hz, 2H), 6.93 (d, *J* = 9.1 Hz, 2H), and 3.82 (s, 3H). ^13^C{1H} NMR (100 MHz, CDCl_3_) δ 164.85, 157.60, 144.42, 140.20, 131.67, 129.08, 128.30, 122.52, 114.74, and 55.78.

*4-Fluorophenyl 4-chlorobenzoate* (**1d**) [69]. Yield 98% (2.45 g). White solid. ^1^H-NMR (400 MHz, CDCl_3_) δ 8.12 (d, *J* = 8.8 Hz, 2H), 7.48 (d, *J* = 8.8 Hz, 2H), 7.19–7.14 (m, 2 H), and 7.14–7.08 (m, 2 H). ^13^C{1H}-NMR (100 MHz, CDCl_3_) δ 164.52, 161.74, 159.31, 146.71, 140.45, 131.70, 129.17, 127.89, 123.20 (d, *J^F^* = 8.4 Hz), and 116.38 (d, *J^F^* = 23.5 Hz). 

*o-Tolyl 4-chlorobenzoate* (**1e**). *New compound.* Yield 97% (2.40 g). Colorless oil. ^1^H-NMR (400 MHz, CDCl_3_) δ 8.13 (d, *J* = 8.7 Hz, 2H), 7.45 (d, *J* = 8.7 Hz, 2H), 7.27–7.09 (m, 4H), and 2.21 (s, 3H). ^13^C{1H}-NMR (100 MHz, CDCl_3_) δ 164.07, 149.45, 140.18, 131.61, 131.32, 130.27, 129.06, 127.97, 127.13, 126.32, 122.00, and 16.31. HRMS (ESI/Q-TOF) *m/z*: [M + Na]^+^ calcd for C_14_H_11_ClO_2_Na 269.0345 found 269.0342. 

*2,6-Dimethylphenyl 4-chlorobenzoate* (**1f**). *New compound.* Yield 98% (2.56 g). Colorless oil. ^1^H-NMR (400 MHz, CDCl_3_) δ 8.16 (d, *J* = 8.5 Hz, 2H), 7.46 (d, *J* = 8.4 Hz, 2H), 7.11–7.04 (m, 3H), and 2.17 (s, 6H). ^13^C{1H}-NMR (100 MHz, CDCl_3_) δ 163.56, 148.30, 140.20, 131.61, 130.32, 129.10, 128.76, 127.80, 126.13, and 16.43. HRMS (ESI/Q-TOF) *m/z*: [M + Na]^+^ calcd for C_15_H_13_ClO_2_Na 283.0502 found 283.0509. 

### 4.5. Characterization Data for Cross-Coupling Products

*Phenyl 4-ethylbenzoate* (Table 2, **2a**) [70]. Prepared according to the general procedure using phenyl 4-chlorobenzoate (0.50 mmol), Fe(acac)_3_ (5 mol%), DMI (200 mol%), THF (0.15 M), and C_2_H_5_MgCl (2.0 M in THF, 1.05 equiv). The reaction mixture was stirred for 180 min at 0 °C. Yield 63% (71.3 mg). White solid. ^1^H-NMR (400 MHz, CDCl_3_) δ 8.12 (d, *J* = 8.4 Hz, 2H), 7.45–7.40 (m, 2H), 7.33 (d, *J* = 8.5 Hz, 2H), 7.29–7.24 (m, 1H), 7.23-7.18 (m, 2H), 2.74 (q, *J* = 7.6 Hz, 2H), and 1.28 (t, *J* = 7.6 Hz, 3H). ^13^C{1H}-NMR (100 MHz, CDCl_3_) δ 165.44, 151.18, 150.78, 130.51, 129.64, 128.29, 127.17, 125.96, 121.95, 29.22, and 15.45.

*4-(Tert-Butyl)phenyl 4-ethylbenzoate* (Table 2, **2b**). *New compound.* Prepared according to the general procedure using 4-(*tert*-butyl)phenyl 4-chlorobenzoate (0.50 mmol), Fe(acac)_3_ (5 mol%), DMI (200 mol%), THF (0.15 M), and C_2_H_5_MgCl (2.0 M in THF, 1.05 equiv). The reaction mixture was stirred for 180 min at 0 °C. Yield 68% (96.1 mg). Colorless oil. ^1^H-NMR (400 MHz, CDCl_3_) δ 8.12 (d, *J* = 8.4 Hz, 2H), 7.43 (d, *J* = 8.8 Hz, 2H), 7.32 (d, *J* = 8.5 Hz, 2H), 7.13 (d, *J* = 8.8 Hz, 2H), 2.74 (q, *J* = 7.6 Hz, 2H), 1.34 (s, 9H), 1.28 (t, *J* = 7.6 Hz, 3H). ^13^C{1H}-NMR (100 MHz, CDCl_3_) δ 165.57, 150.66, 148.81, 148.73, 130.49, 128.25, 127.31, 126.53, 121.21, 34.66, 31.61, 29.21, and 15.46. HRMS (ESI/Q-TOF) *m/z*: [M + Na]^+^ calcd for C_19_H_22_O_2_Na 305.1518 found 305.1519.

*4-Methoxyphenyl 4-ethylbenzoate* (Table 2, **2c**). *New compound.* Prepared according to the general procedure using 4-methoxyphenyl 4-chlorobenzoate (0.50 mmol), Fe(acac)_3_ (5 mol%), DMI (200 mol%), THF (0.15 M), and C_2_H_5_MgCl (2.0 M in THF, 1.05 equiv). The reaction mixture was stirred for 180 min at 0 °C. Yield 81% (103.8 mg). White solid. Mp = 101–103 °C. ^1^H-NMR (400 MHz, CDCl_3_) δ 8.11 (d, *J* = 8.3 Hz, 2H), 7.32 (d, *J* = 8.5 Hz, 2H), 7.12 (d, *J* = 9.1 Hz, 2H), 6.93 (d, *J* = 9.1 Hz, 2H), 3.82 (s, 3H), 2.74 (q, *J* = 7.6 Hz, 2H), 1.28 (t, *J* = 7.6 Hz, 3H). ^13^C{1H}-NMR (100 MHz, CDCl_3_) δ 165.79, 157.39, 150.67, 144.63, 130.46, 128.25, 127.22, 122.67, 114.65, 55.77, 29.21, and 15.44. HRMS (ESI/Q-TOF) *m/z*: [M + Na]^+^ calcd for C_16_H_16_O_3_Na 279.0997 found 279.0997.

*4-Fluorophenyl 4-ethylbenzoate* (Table 2, **2d**). *New compound.* Prepared according to the general procedure using 4-fluorophenyl 4-chlorobenzoate (0.50 mmol), Fe(acac)_3_ (5 mol%), DMI (200 mol%), THF (0.15 M), and C_2_H_5_MgCl (2.0 M in THF, 1.05 equiv). The reaction mixture was stirred for 180 min at 0 °C. Yield 51% (62.4 mg). White solid. Mp = 38–40 °C. ^1^H-NMR (400 MHz, CDCl_3_) δ 8.11 (d, *J* = 8.4 Hz, 2H), 7.33 (d, *J* = 8.1 Hz, 2H), 7.19–7.14 (m, 2H), 7.13–7.07 (m, 2H), 2.75 (q, *J* = 7.6 Hz, 2H), 1.28 (t, *J* = 7.6 Hz, 3H). ^13^C{1H}-NMR (100 MHz, CDCl_3_) δ 165.45, 161.63, 159.20, 150.96, 146.97 (d, *J^F^* = 2.9 Hz), 130.51, 128.33, 126.86, 123.33 (d, *J^F^* = 8.5 Hz), 116.28 (d, *J^F^* = 23.5 Hz), 29.23, and 15.43. HRMS (ESI/Q-TOF) *m/z*: [M + Na]^+^ calcd for C_15_H_13_FO_2_Na 267.0797 found 267.0794.

*o-Tolyl 4-ethylbenzoate* (Table 2, **2e**). *New compound.* Prepared according to the general procedure using *o*-tolyl 4-chlorobenzoate (0.50 mmol), Fe(acac)_3_ (5 mol%), DMI (200 mol%), THF (0.15 M), and C_2_H_5_MgCl (2.0 M in THF, 1.05 equiv). The reaction mixture was stirred for 180 min at 0 °C. Yield 80% (96.1 mg). Colorless oil. ^1^H-NMR (400 MHz, CDCl_3_) δ 8.14 (d, *J* = 8.3 Hz, 2H), 7.34 (d, *J* = 8.4 Hz, 2H), 7.30–7.22 (m, 2H), 7.20–7.11 (m, 2H), 2.75 (q, *J* = 7.6 Hz, 2H), 2.23 (s, 3H), and 1.29 (t, *J* = 7.6 Hz, 3H). ^13^C{1H}-NMR (100 MHz, CDCl_3_) δ 165.10, 150.77, 149.76, 131.31, 130.51, 128.32, 127.12, 127.09, 126.17, 122.23, 29.23, 16.43, and 15.47. HRMS (ESI/Q-TOF) *m/z*: [M + Na]^+^ calcd for C_16_H_16_O_2_Na 263.1048 found 263.1044.

*2,6-Dimethylphenyl 4-ethylbenzoate* (Table 2, **2f**). *New compound.* Prepared according to the general procedure using 2,6-dimethylphenyl 4-chlorobenzoate (0.50 mmol), Fe(acac)_3_ (5 mol%), DMI (200 mol%), THF (0.15 M), and C_2_H_5_MgCl (2.0 M in THF, 1.05 equiv). The reaction mixture was stirred for 180 min at 0 °C. Yield 90% (114.2 mg). Colorless oil. ^1^H-NMR (400 MHz, CDCl_3_) δ 8.17 (d, *J* = 8.3 Hz, 2H), 7.34 (d, *J* = 8.4 Hz, 2H), 7.12–7.06 (m, 3H), 2.74 (q, *J* = 7.6 Hz, 2H), 2.19 (s, 6H), and 1.29 (t, *J* = 7.6 Hz, 3H). ^13^C{1H}-NMR (100 MHz, CDCl_3_) δ 164.56, 150.73, 148.53, 130.54, 130.50, 128.72, 128.32, 126.88, 125.97, 29.20, 16.54, and 15.44. HRMS (ESI/Q-TOF) *m/z*: [M + Na]^+^ calcd for C_17_H_18_O_2_Na 277.1205 found 277.1209.

*4-Methoxyphenyl 4-hexylbenzoate* (Table 2, **2g**). *New compound.* Prepared according to the general procedure using 4-methoxyphenyl 4-chlorobenzoate (0.50 mmol), Fe(acac)_3_ (5 mol%), DMI (200 mol%), THF (0.15 M), and C_6_H_13_MgCl (2.0 M in THF, 1.05 equiv). The reaction mixture was stirred for 180 min at 0 °C. Yield 83% (129.8 mg). White solid. Mp = 64–66 °C. ^1^H-NMR (400 MHz, CDCl_3_) δ 8.10 (d, *J* = 8.3 Hz, 2H), 7.30 (d, *J* = 8.4 Hz, 2H), 7.12 (d, *J* = 9.1 Hz, 2H), 6.93 (d, *J* = 9.1 Hz, 2H), 3.82 (s, 3H), 2.69 (t, *J* = 7.6 Hz, 2H), 1.70–1.60 (m, 2H), 1.37–1.28 (m, 6H), and 0.89 (t, *J* = 6.9 Hz, 3H). ^13^C{1H}-NMR (100 MHz, CDCl_3_) δ 165.81, 157.40, 149.45, 144.65, 130.37, 128.80, 127.20, 122.67, 114.65, 55.78, 36.26, 31.84, 31.30, 29.09, 22.76, and 14.27. HRMS (ESI/Q-TOF) *m/z*: [M + Na]^+^ calcd for C_20_H_24_O_3_Na 335.1623 found 335.1614.

*4-Methoxyphenyl 4-tetradecylbenzoate* (Table 2, **2h**). *New compound.* Prepared according to the general procedure using 4-methoxyphenyl 4-chlorobenzoate (0.50 mmol), Fe(acac)_3_ (5 mol%), DMI (200 mol%), THF (0.15 M), and C_14_H_29_MgCl (1.0 M in THF, 1.05 equiv). The reaction mixture was stirred for 180 min at 0 °C. Yield 76% (161.7 mg). White solid. Mp = 63–65 °C. ^1^H-NMR (400 MHz, CDCl_3_) δ 8.10 (d, *J* = 8.3 Hz, 2H), 7.30 (d, *J* = 8.3 Hz, 2H), 7.12 (d, *J* = 9.1 Hz, 2H), 6.93 (d, *J* = 9.1 Hz, 2H), 3.82 (s, 3H), 2.69 (t, *J* = 7.7 Hz, 2H), 1.69–1.60 (m, 2H), 1.35–1.24 (m, 22H), and 0.88 (t, *J* = 6.8 Hz, 3H). ^13^C{1H}-NMR (100 MHz, CDCl_3_) δ 165.80, 157.39, 149.46, 144.64, 130.37, 128.79, 127.19, 122.67, 114.64, 55.77, 36.26, 32.11, 31.35, 29.84, 29.75, 29.65, 29.55, 29.44, 22.88, and 14.32. HRMS (ESI/Q-TOF) *m/z*: [M + H]^+^ calcd for C_28_H_41_O_3_ 425.3056 found 425.3056.

*4-Methoxyphenyl 4-cyclohexylbenzoate* (Table 2, **2i**). *New compound.* Prepared according to the general procedure using 4-methoxyphenyl 4-chlorobenzoate (0.50 mmol), Fe(acac)_3_ (5 mol%), DMI (200 mol%), THF (0.15 M), and *c*-C_6_H_11_MgCl (1.0 M in THF, 1.20 equiv). The reaction mixture was stirred for 15 h at 0 °C. Yield 37% (57.8 mg). White solid. Mp = 131–133 °C. ^1^H-NMR (400 MHz, CDCl_3_) δ 8.11 (d, *J* = 8.4 Hz, 2H), 7.33 (d, *J* = 8.2 Hz, 2H), 7.11 (d, *J* = 9.1 Hz, 2H), 6.93 (d, *J* = 9.1 Hz, 2H), 3.82 (s, 3H), 2.65–2.54 (m, 1H), 1.94–1.82 (m, 4H), 1.81–1.73 (m, 1H), 1.51–1.34 (m, 4H), and 1.33–1.23 (m, 1H). ^13^C{1H}-NMR (100 MHz, CDCl_3_) δ 165.77, 157.38, 154.38, 144.64, 130.45, 127.31, 127.23, 122.67, 114.64, 55.77, 44.93, 34.30, 26.89, and 26.19. HRMS (ESI/Q-TOF) *m/z*: [M + Na]^+^ calcd for C_20_H_22_O_3_Na 333.1467 found 333.1474.

*4-Methoxyphenyl 4-phenethylbenzoate* (Table 2, **2j**). *New compound.* Prepared according to the general procedure using 4-methoxyphenyl 4-chlorobenzoate (0.50 mmol), Fe(acac)_3_ (5 mol%), DMI (200 mol%), THF (0.15 M), and PhCH_2_CH_2_MgCl (1.0 M in THF, 1.2 equiv). The reaction mixture was stirred for 15 h at 0 °C. Yield 82% (136.1 mg). White solid. Mp = 116–118 °C. ^1^H-NMR (400 MHz, CDCl_3_) δ 8.09 (d, *J* = 8.3 Hz, 2H), 7.31–7.26 (m, 4H), 7.22–7.14 (m, 3H), 7.11 (d, *J* = 9.1 Hz, 2H), 6.93 (d, *J* = 9.1 Hz, 2H), 3.80 (s, 3H), 3.04–2.98 (m, 2H), and 2.97–2.92 (m, 2H). ^13^C{1H}-NMR (100 MHz, CDCl_3_) δ 165.71, 157.38, 148.03, 144.58, 141.19, 130.40, 128.88, 128.61, 128.57, 127.49, 126.27, 122.63, 114.63, 55.73, 38.07, and 37.59. HRMS (ESI/Q-TOF) *m/z*: [M + Na]^+^ calcd for C_22_H_20_O_3_Na 355.1310 found 355.1308.

## Figures and Tables

**Table 1:**
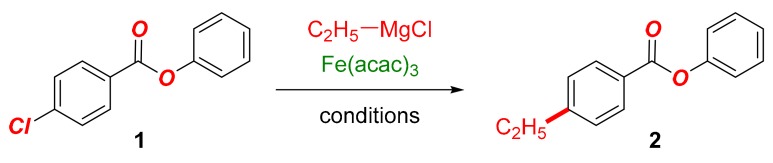
Optimization of iron-catalyzed cross-coupling.^1^ DMI: 1,3-dimethyl-2-imidazolidinone.

Entry	Fe(acac)_3_ (mol%)	Ligand	mol %	Addition Time (min)	Time (min)	Yield (%) ^2^
1 ^3^	5	DMI	200	0	10	27
2	5	DMI	200	0	10	27
3	5	DMI	200	60	180	65
4	5	DMI	600	60	180	52
5	5	DMI	20	60	180	48
6	5	-	-	60	180	44
7 ^4^	5	DMI	200	60	180	57
8	5	DMI	200	180	60	52
9	10	DMI	200	60	180	60
10	50	DMI	200	60	180	28
11 ^5^	5	DMI	200	0	180	52
12 ^6^	5	DMI	200	0	180	<10
13	-	-	-	60	180	0
14	-	DMI	200	60	180	0
15	5	NMP	200	60	180	57

^1^ Conditions: **1** (0.50 mmol), Fe(acac)_3_ (5 mol%), THF (0.15 M), C_2_H_5_MgCl (1.05 equiv, 2.0 M, THF), 0 °C, 180 min. RMgCl added dropwise over 60 min. ^2^ Yields determined by ^1^H-NMR and/or GC-MS. ^3^ C_2_H_5_MgCl (1.20 equiv). ^4^ C_2_H_5_MgCl (0.83 equiv). ^5^ −40 °C. ^6^ −78 °C.

**Table 2:**
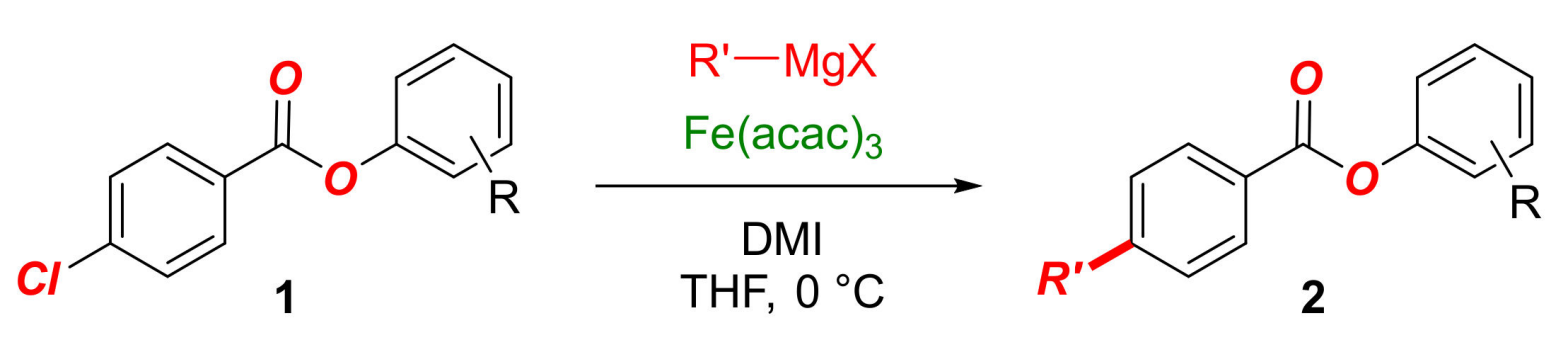
Iron-catalyzed C(sp^2^)–C(sp^3^) cross-coupling of aryl chlorobenzoates with alkyl Grignards.^1.^

Entry	Substrate	2	Product	Yield (%)
1	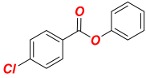	**2a**	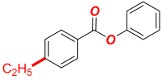	63
2	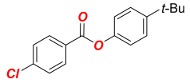	**2b**	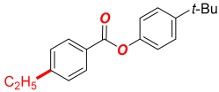	68
3	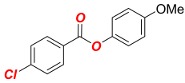	**2c**	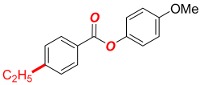	81
4	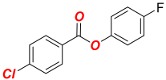	**2d**	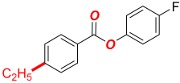	51
5	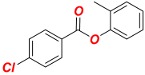	**2e**	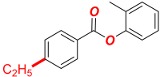	80
6	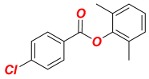	**2f**	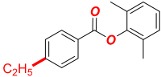	90
7	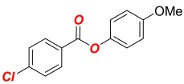	**2g**	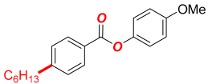	83
8	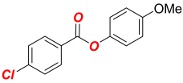	**2h**	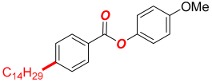	76
9 ^2^	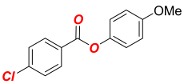	**2i**	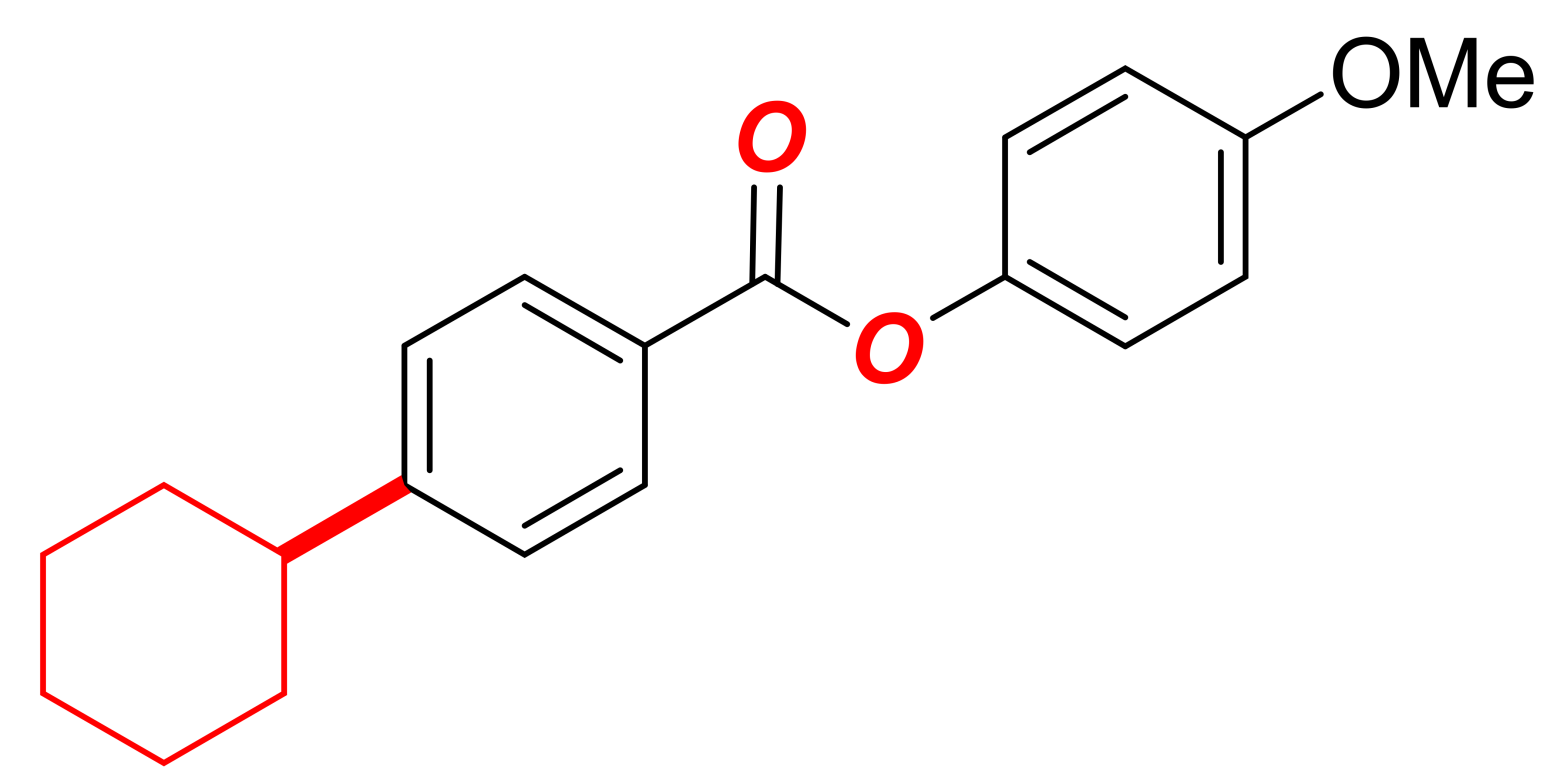	37
10 ^2^	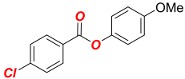	**2j**	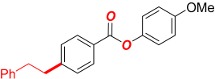	82

^1^ Conditions: **1** (0.50 mmol), Fe(acac)_3_ (5 mol%), THF (0.15 M), DMI (200 mol%), RMgX (1.05 equiv, THF), 0 °C, 180 min. RMgX added dropwise over 60 min. Isolated yields. ^2^ RMgX (1.20 equiv), 15 h. See the Supplementary Materials for details.

**Table 3:**
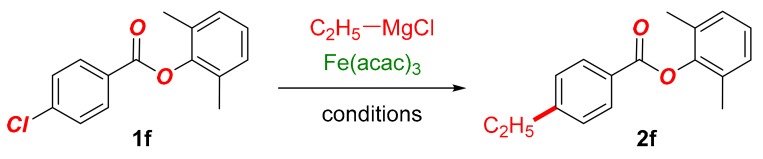
Ligand effect on iron-catalyzed cross-coupling of aryl chlorobenzoates: cross-coupling vs. nucleophilic addition.^1.^ DMPU: 1,3-dimethyl-3,4,5,6-tetrahydro-2(1*H*)-pyrimidinone); NMP: *N*-methyl-2-pyrrolidone, TMU: 1,1,3,3-tetramethylurea.

Entry	Fe(acac)_3_ (mol%)	Ligand	mol%	Time (min)	Yield (%) ^2^
1	5	DMI	200	180	98 (2)
2	5	DMPU	200	180	>98 (1)
3	5	TMU	200	180	>98 (<1)
4	5	NMP	200	180	95 (4)
5	5	*N*-Methylcaprolactam	200	180	92 (7)
6 ^3^	5	Bis(OMeEt)-BA	200	180	57 (<1)
7 ^4^	5	Pip-BA	200	180	75 (<1)

^1^ Conditions: **1** (0.50 mmol), Fe(acac)_3_ (5 mol%), THF (0.15 M), C_2_H_5_MgCl (1.0 equiv, 2.0 M, THF), 0 °C, 180 min. RMgCl added dropwise over 60 min. ^2^ Determined by ^1^H-NMR and/or GC-MS. The number in brackets corresponds to the alcohol addition product. Entries 6–7: **1**: 43% and 25%. ^3^ Ligand: *N*,*N*-bis(2-methoxyethyl)benzamide. ^4^ Ligand: Phenyl(piperidin-1-yl)methanone. See Figure 1 for structures.

## Supplementary Materials

^1^H and ^13^C-NMR spectra are available online at https://www.mdpi.com/1420-3049/25/1/230/s1.
