# Supplementary material for: Iron-Catalyzed C(sp2)–C(sp3) Cross-Coupling of Aryl Chlorobenzoates with Alkyl Grignard Reagents
Source: Molecules. 2020 Jan 6;25(1):230. doi: 10.3390/molecules25010230 (PMC6983197; doi:10.3390/molecules25010230)
Supplement: Supplementary file 1 [file molecules-25-00230-s001.pdf]

Iron-Catalyzed C(sp^2)–C(sp^3) Cross-Coupling of Aryl Chlorobenzoates with Alkyl Grignard Reagents

Elwira Bisz,*,† and Michal Szostak*,†,‡

†*Department of Chemistry, Opole University, 48 Oleska Street, 45-052 Opole, Poland*
‡*Department of Chemistry, Rutgers University, 73 Warren Street, Newark, NJ 07102, USA*

ebisz@uni.opole.pl; michal.szostak@rutgers.edu

Supporting Information

Table of Contents S1

^{1}H and ^{13}C NMR Spectra S2

Corresponding Author:
Dr. E. Bisz
Department of Chemistry, Opole University
48 Oleska Street, 45-052 Opole, Poland
E-mail: ebisz@uni.opole.pl

Prof. Dr. M. Szostak
Department of Chemistry, Rutgers University
73 Warren Street, Newark, NJ 07102, United States
E-mail: michal.szostak@rutgers.edu

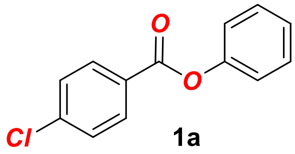
O
O
Cl

1a

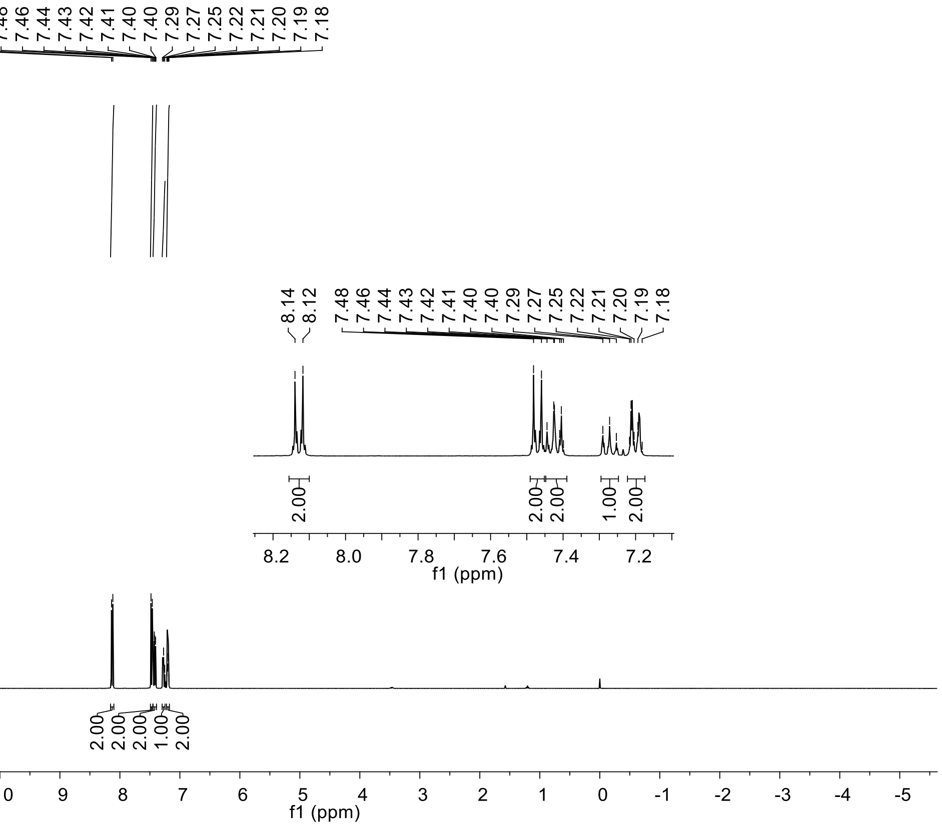
8.14
8.12
7.48
7.46
7.44
7.43
7.42
7.41
7.40
7.40
7.29
7.27
7.25
7.22
7.21
7.20
7.19
7.18
2.00
2.00
2.00
1.00
2.00
f1 (ppm)

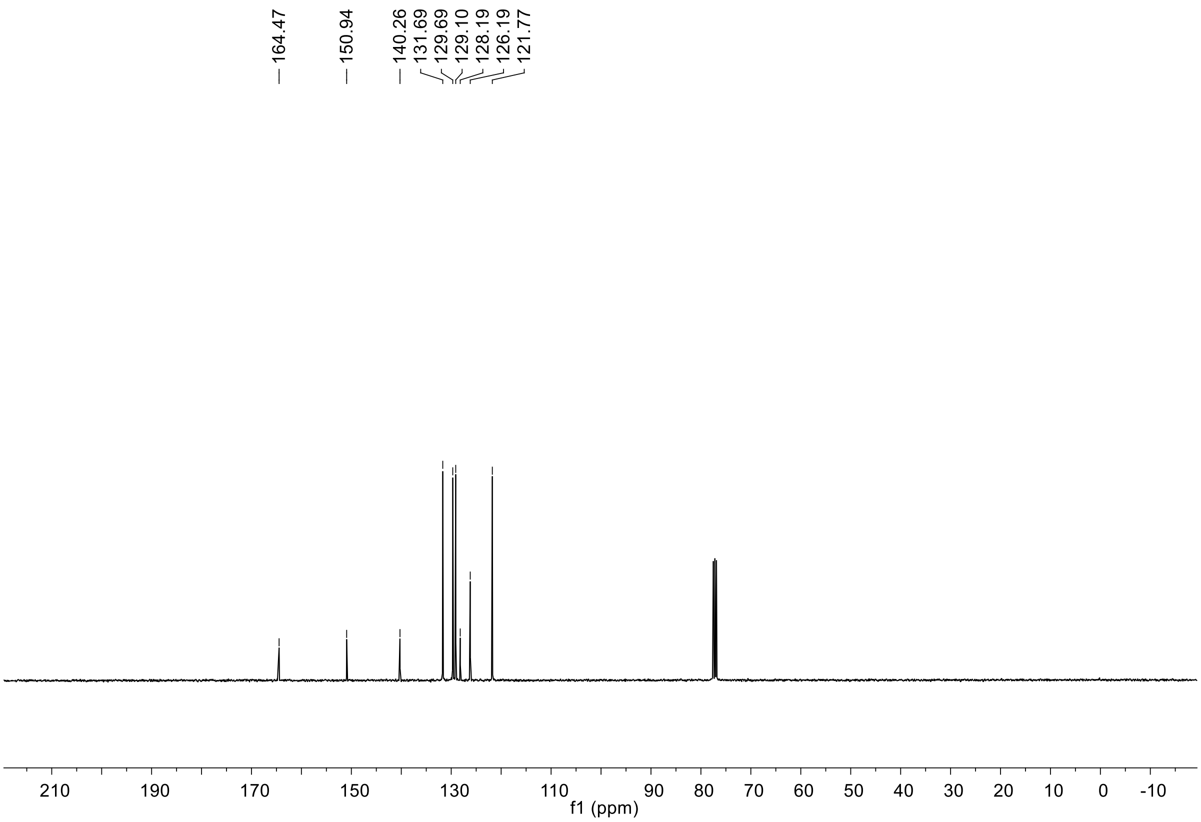
164.47
150.94
140.26
131.69
129.69
129.10
128.19
126.19
121.77
f1 (ppm)

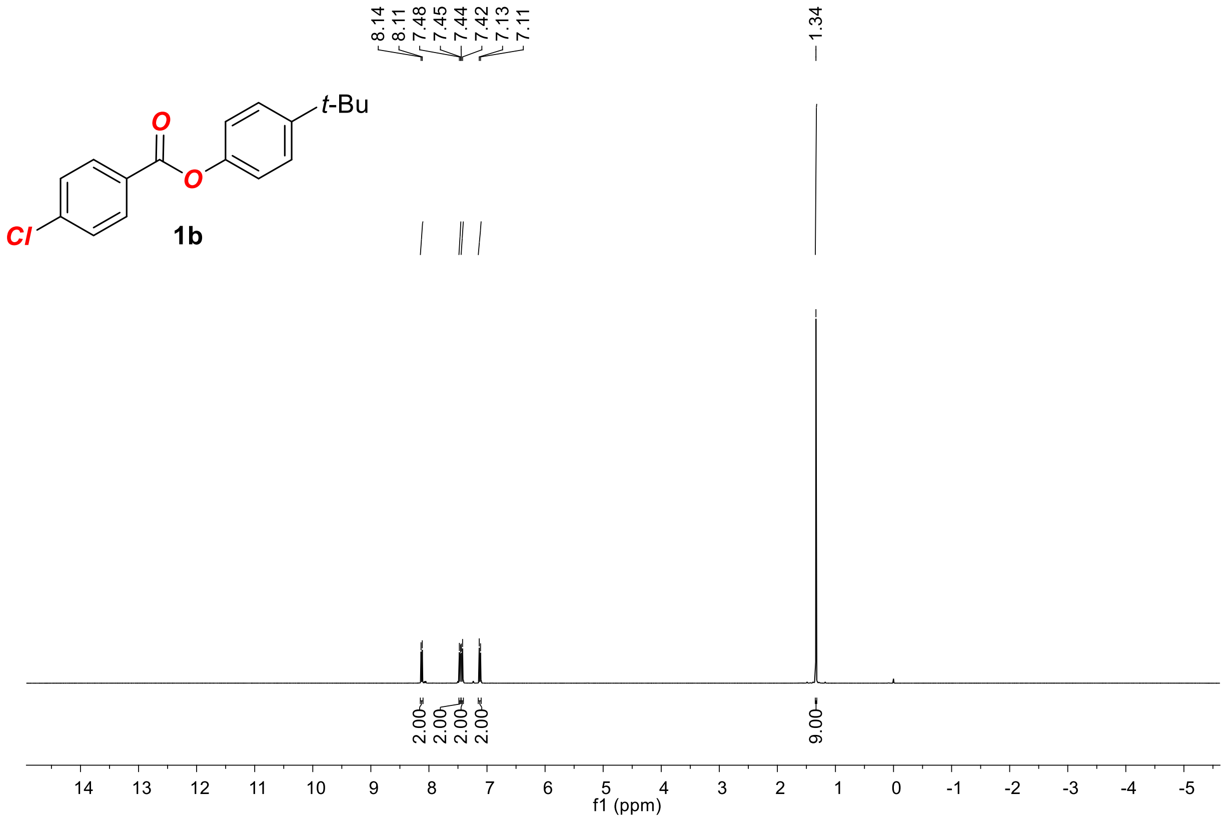
t-Bu
O
O
Cl
1b
8.14
8.11
7.48
7.45
7.44
7.42
7.13
7.11
1.34
2.00
2.00
2.00
2.00
9.00
14
13
12
11
10
9
8
7
6
5
4
3
2
1
0
-1
-2
-3
-4
-5
f1 (ppm)

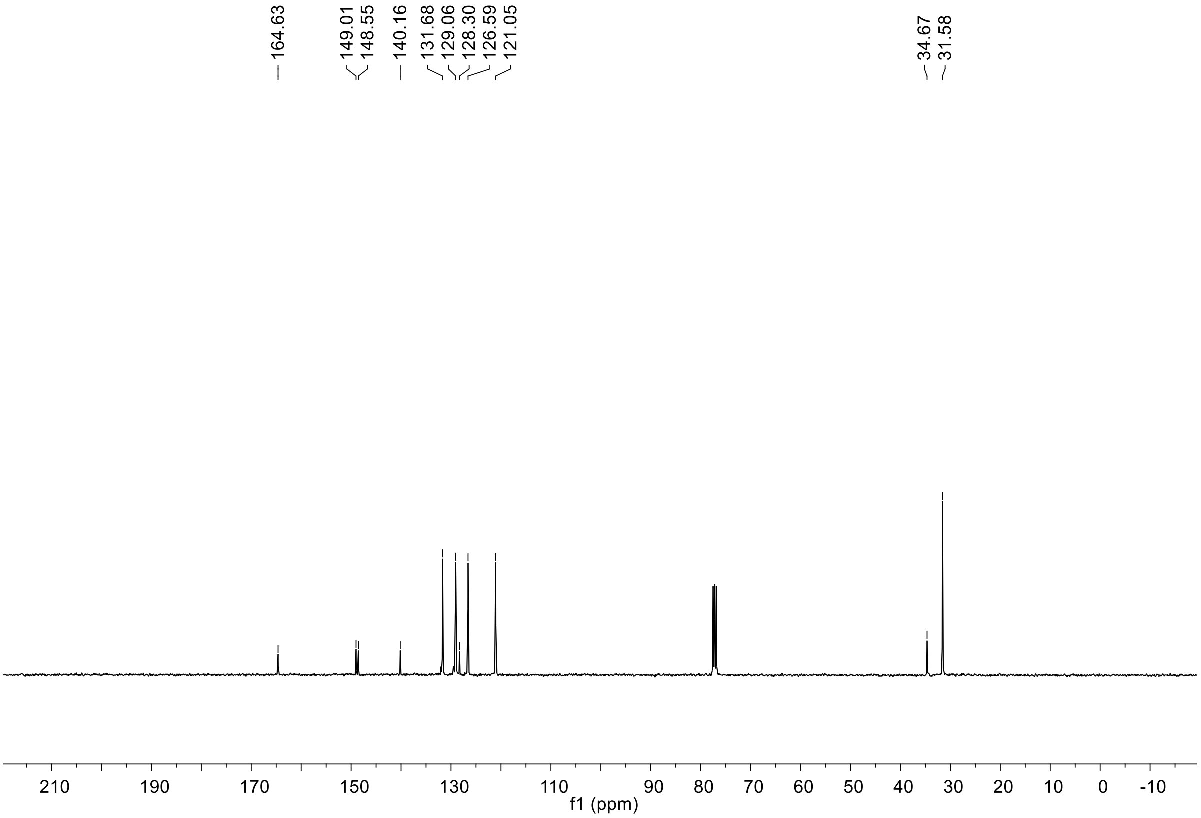
164.63
149.01
148.55
140.16
131.68
129.06
128.30
126.59
121.05
34.67
31.58
210
190
170
150
130
110
90
80
70
60
50
40
30
20
10
0
-10
f1 (ppm)

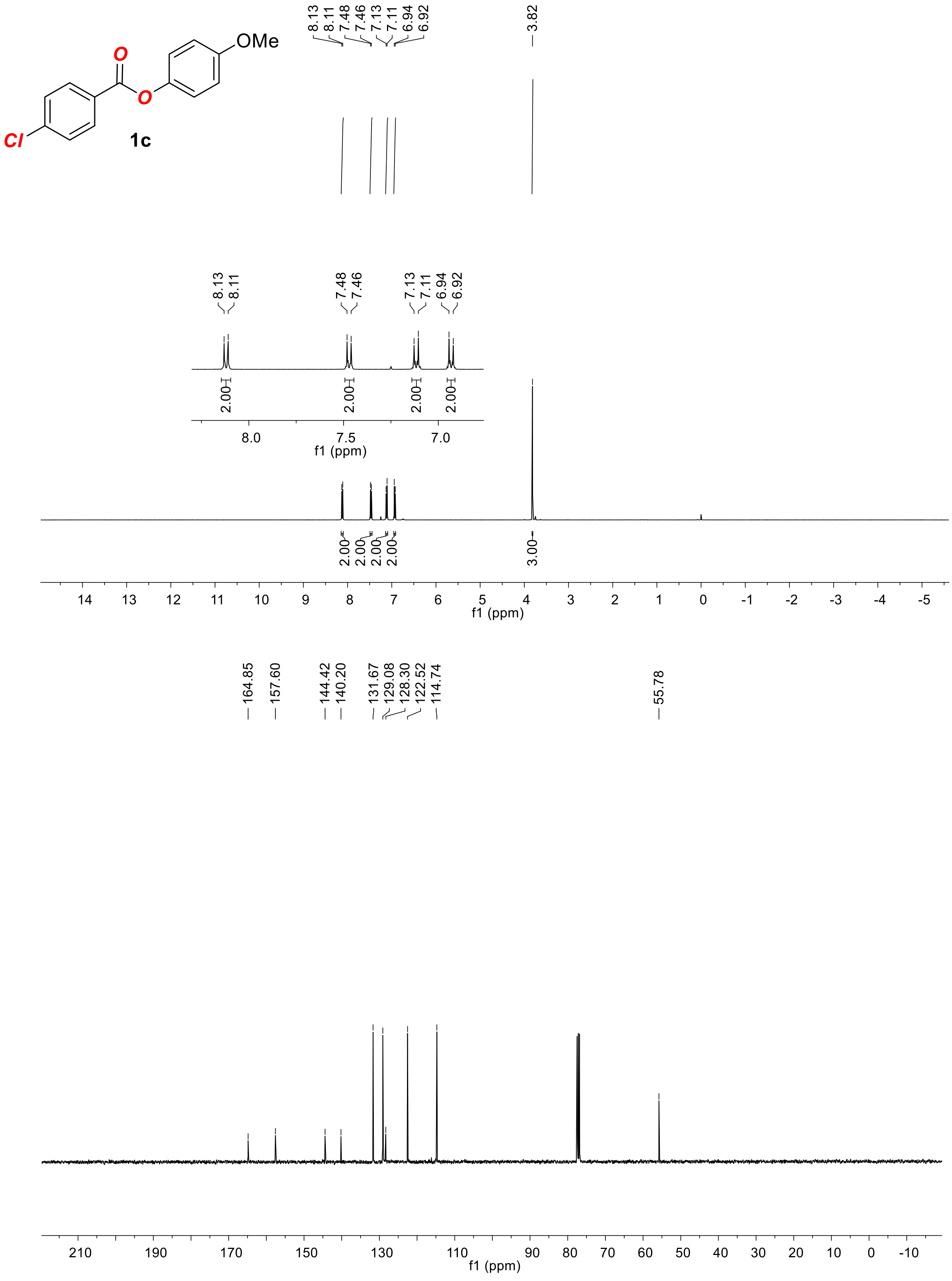
OMe
O
O
Cl
1c
8.13
8.11
7.48
7.46
7.13
7.11
6.94
6.92
3.82
2.00
3.00
f1 (ppm)
164.85
157.60
144.42
140.20
131.67
129.08
128.30
122.52
114.74
55.78

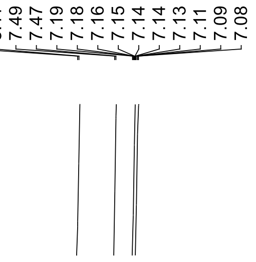
8.13
8.11
7.49
7.47
7.19
7.18
7.16
7.15
7.14
7.14
7.13
7.11
7.09
7.08

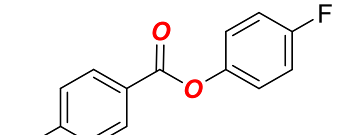
O
O
F
Cl

1d

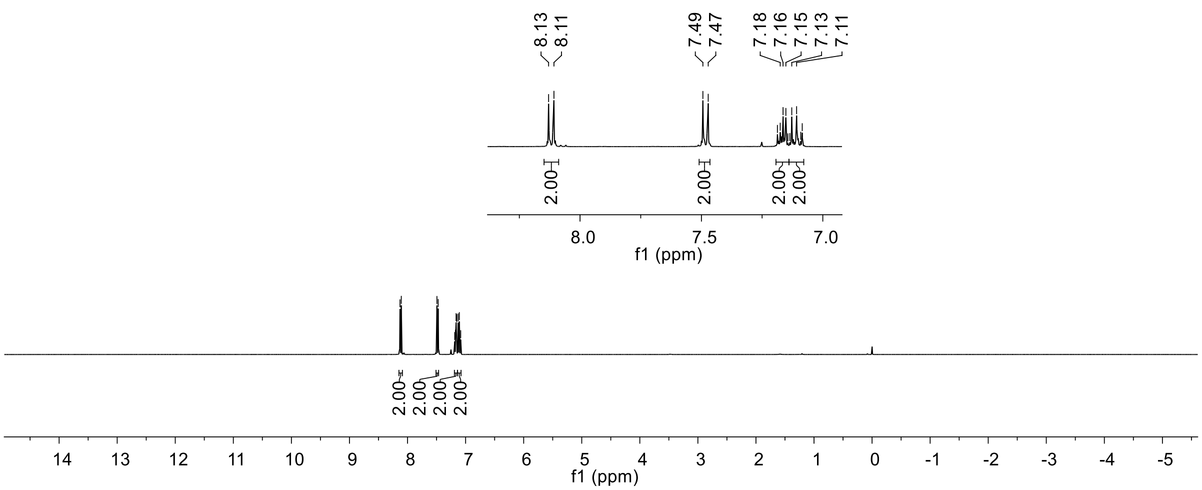
8.13
8.11
7.49
7.47
7.18
7.16
7.15
7.13
7.11
2.00
2.00
2.00
2.00
8.0
7.5
7.0
f1 (ppm)
14
13
12
11
10
9
8
7
6
5
4
3
2
1
0
-1
-2
-3
-4
-5
f1 (ppm)

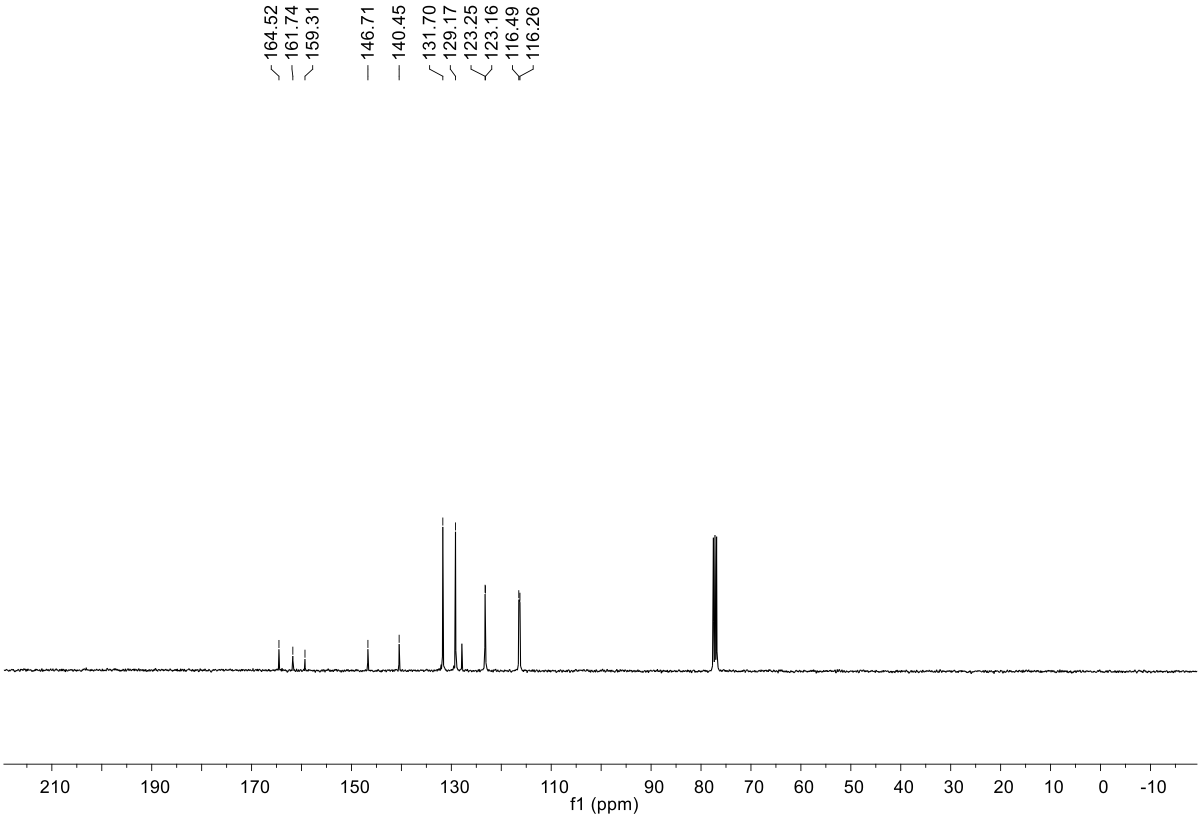
164.52
161.74
159.31
146.71
140.45
131.70
129.17
123.25
123.16
116.49
116.26
210
190
170
150
130
110
90
80
70
60
50
40
30
20
10
0
-10
f1 (ppm)

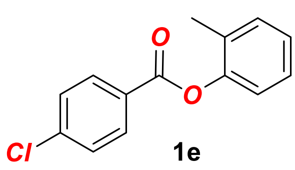
O
O
Cl

1e

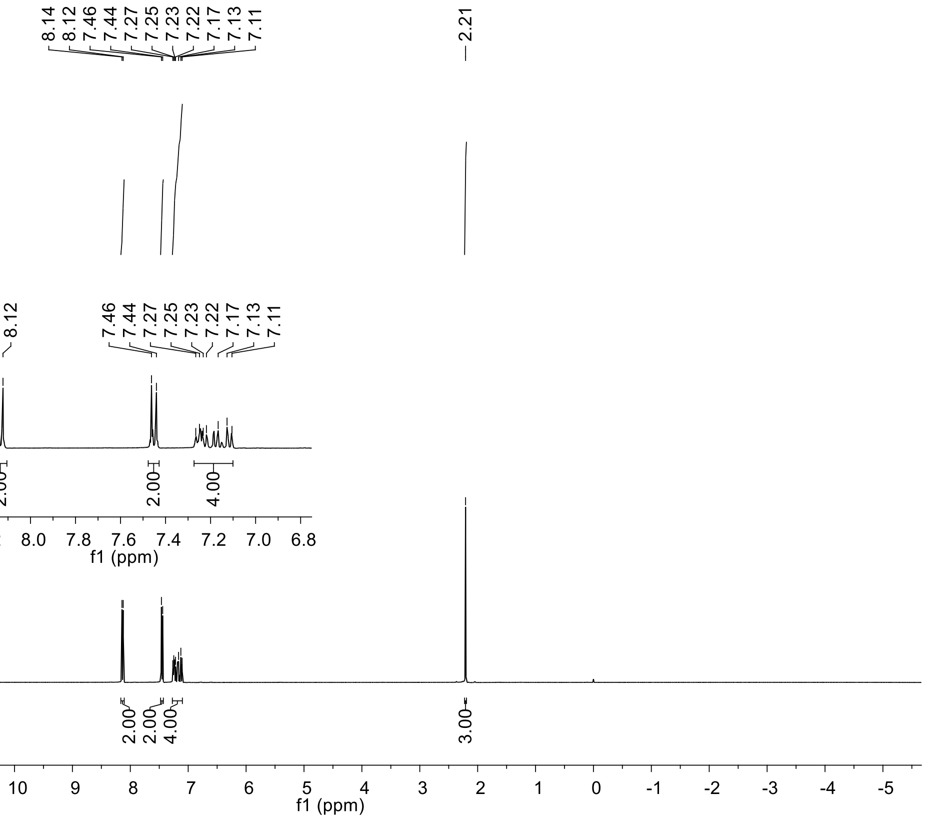
8.14
8.12
7.46
7.44
7.27
7.25
7.23
7.22
7.17
7.13
7.11
2.21
2.00
2.00
4.00
3.00
f1 (ppm)

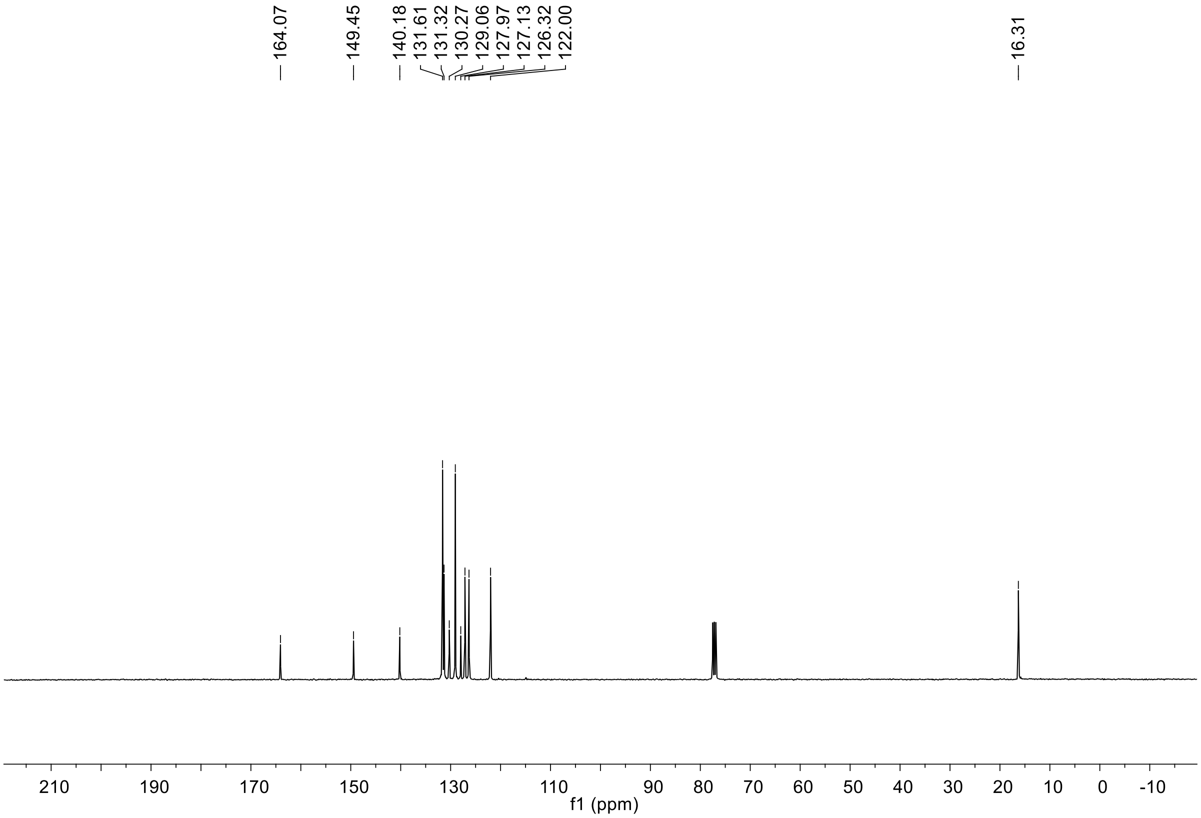
164.07
149.45
140.18
131.61
131.32
130.27
129.06
127.97
127.13
126.32
122.00
16.31
f1 (ppm)

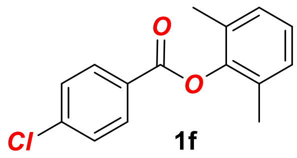
O
O
Cl

1f

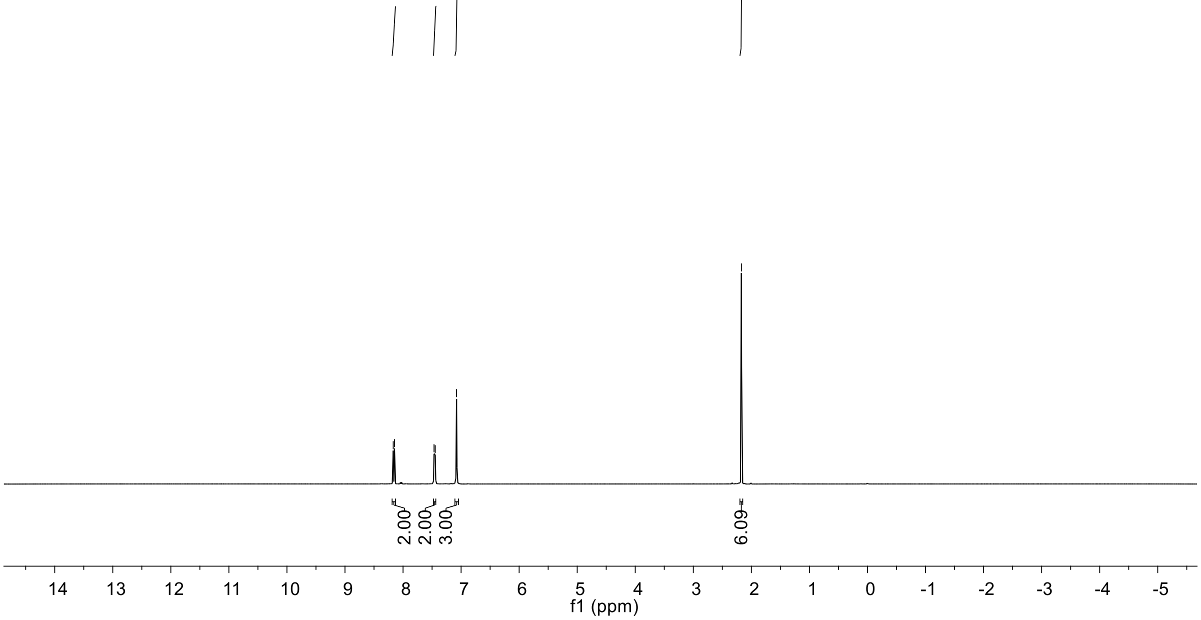
8.17
8.15
7.47
7.45
7.08
7.07
2.17
2.00
2.00
3.00
6.09
14
13
12
11
10
9
8
7
6
5
4
3
2
1
0
-1
-2
-3
-4
-5
f1 (ppm)

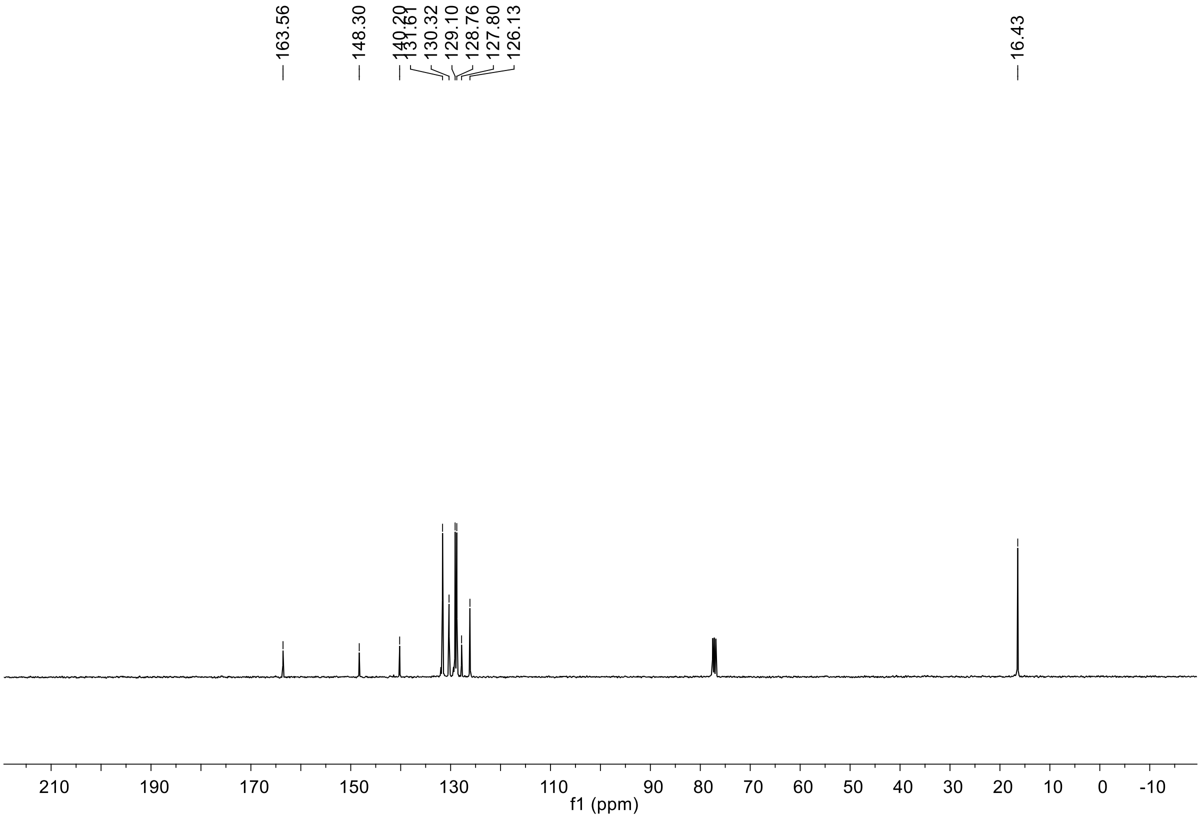
163.56
148.30
140.20
131.61
130.32
129.10
128.76
127.80
126.13
16.43
210
190
170
150
130
110
90
80
70
60
50
40
30
20
10
0
-10
f1 (ppm)

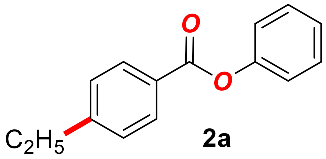
O
O
C2H5

2a

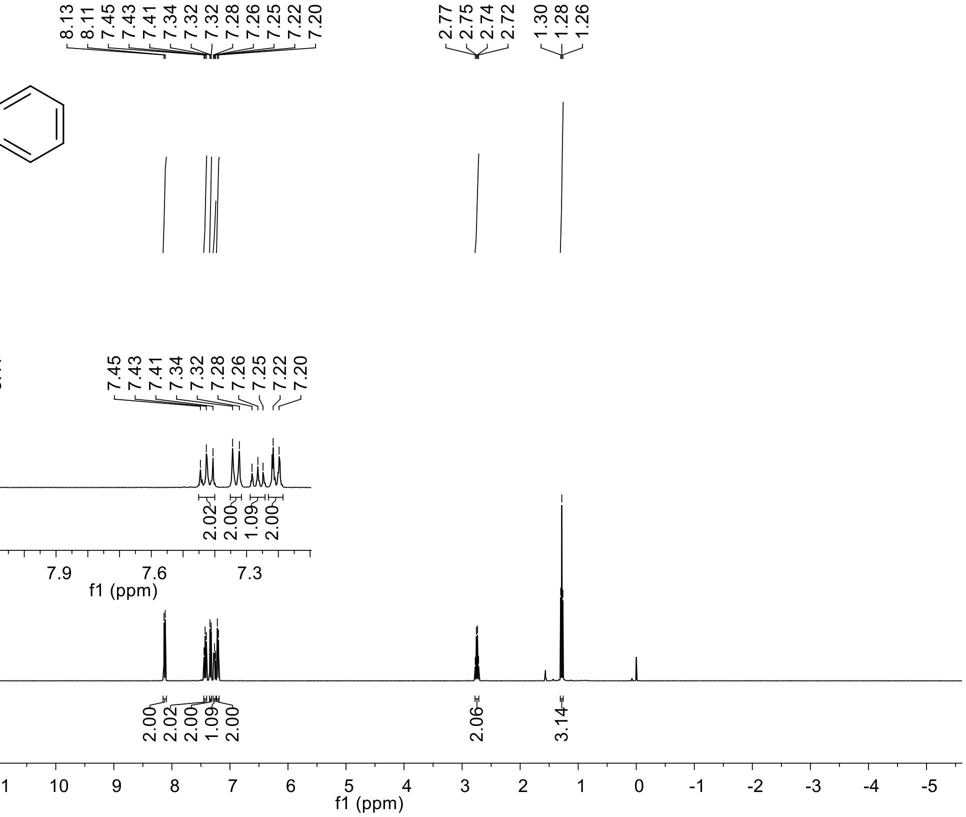
8.13 8.11 7.45 7.43 7.41 7.34 7.32 7.28 7.26 7.25 7.22 7.20
2.77 2.75 2.74 2.72 1.30 1.28 1.26
2.00 2.02 2.00 1.09 2.00
2.06 3.14
f1 (ppm)

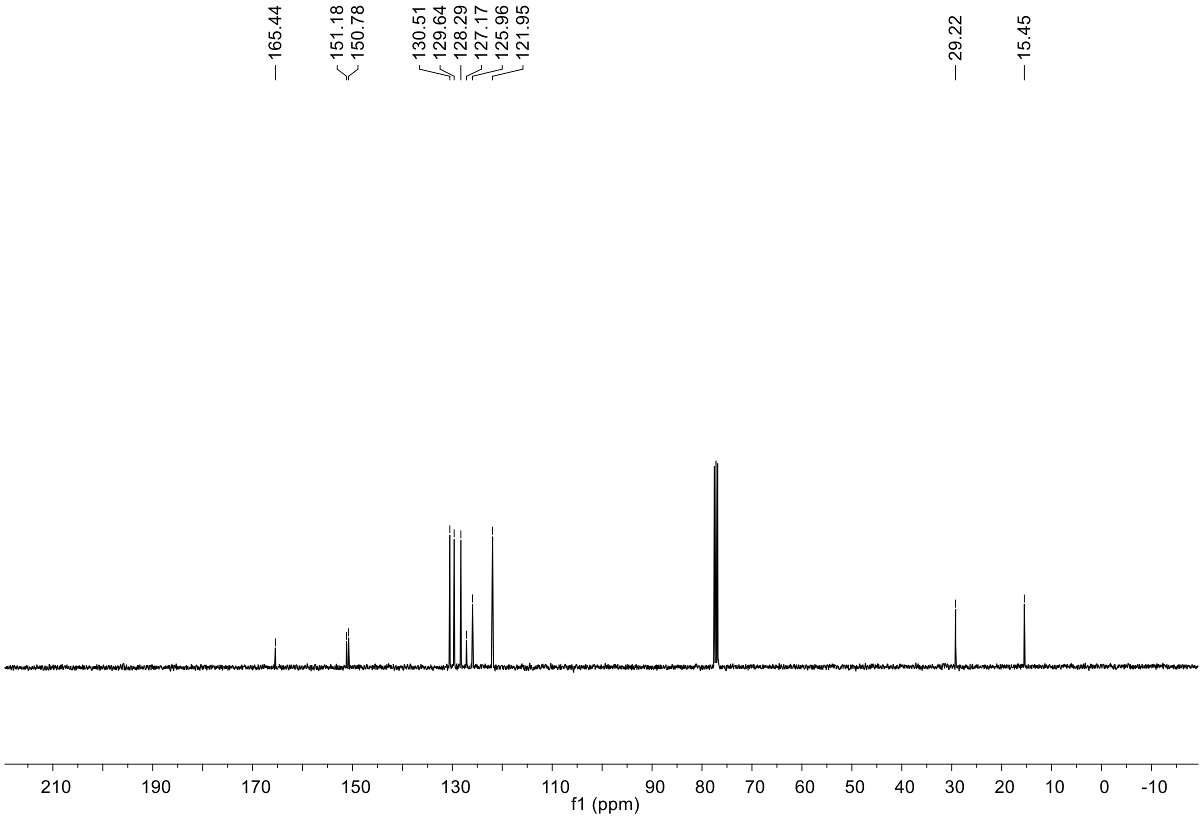
165.44
151.18 150.78
130.51 129.64 128.29 127.17 125.96 121.95
29.22
15.45
f1 (ppm)

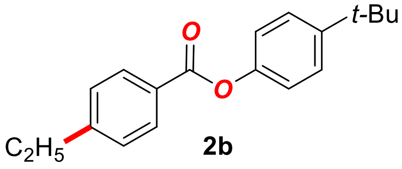
O
O
t-Bu
C2H5

2b

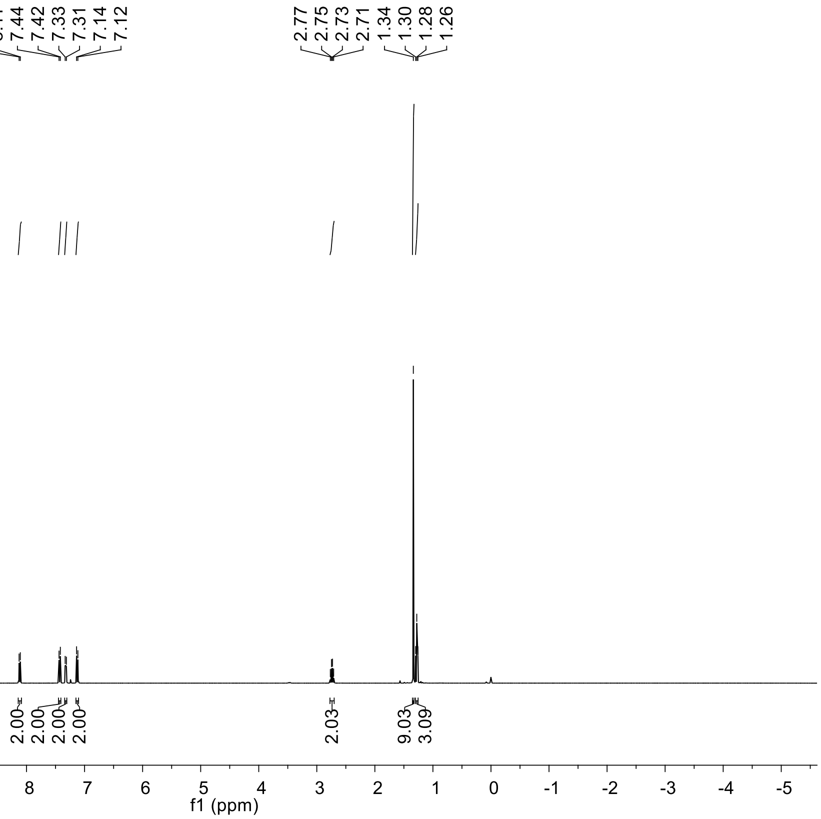
8.13
8.11
7.44
7.42
7.33
7.31
7.14
7.12
2.77
2.75
2.73
2.71
1.34
1.30
1.28
1.26
2.00
2.00
2.00
2.00
2.03
9.03
3.09
14 13 12 11 10 9 8 7 6 5 4 3 2 1 0 -1 -2 -3 -4 -5
f1 (ppm)

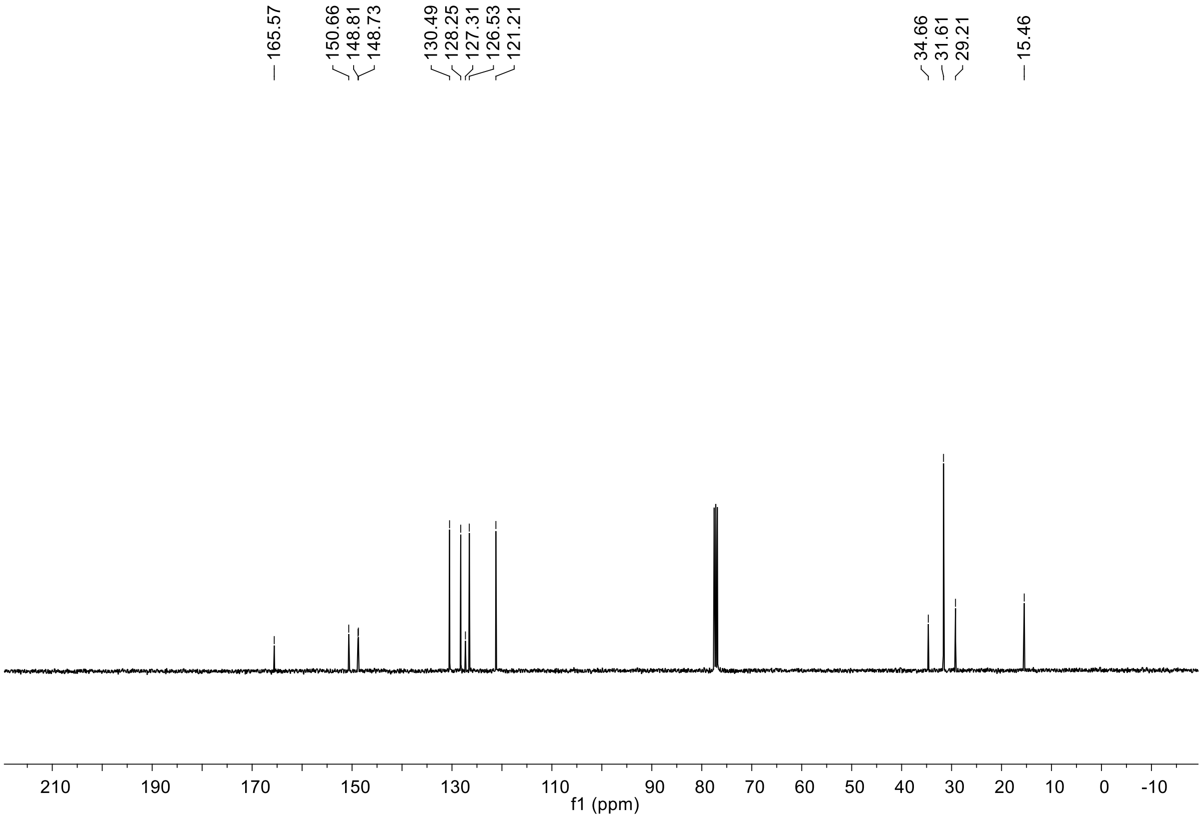
165.57
150.66
148.81
148.73
130.49
128.25
127.31
126.53
121.21
34.66
31.61
29.21
15.46
210 190 170 150 130 110 90 80 70 60 50 40 30 20 10 0 -10
f1 (ppm)

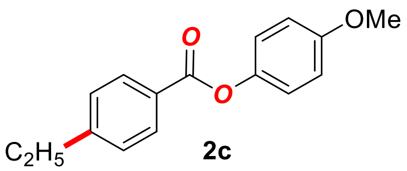
OMe
O
O
C_2H_5

2c

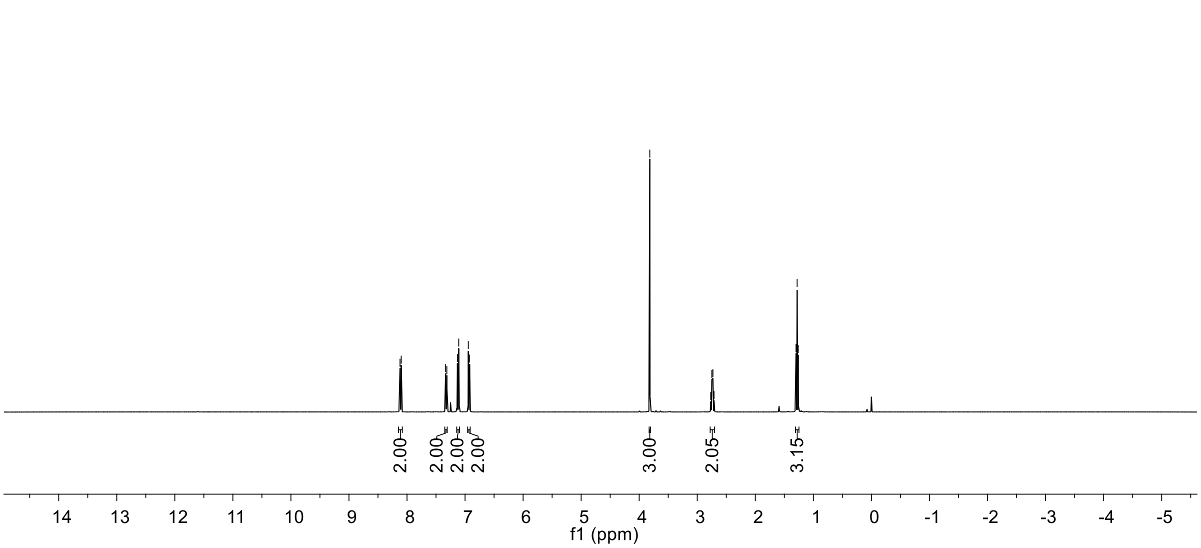
8.12
8.10
7.33
7.31
7.13
7.11
6.94
6.92
3.82
2.77
2.75
2.73
2.71
1.30
1.28
1.26
2.00
2.00
2.00
2.00
3.00
2.05
3.15
14 13 12 11 10 9 8 7 6 5 4 3 2 1 0 -1 -2 -3 -4 -5
f1 (ppm)

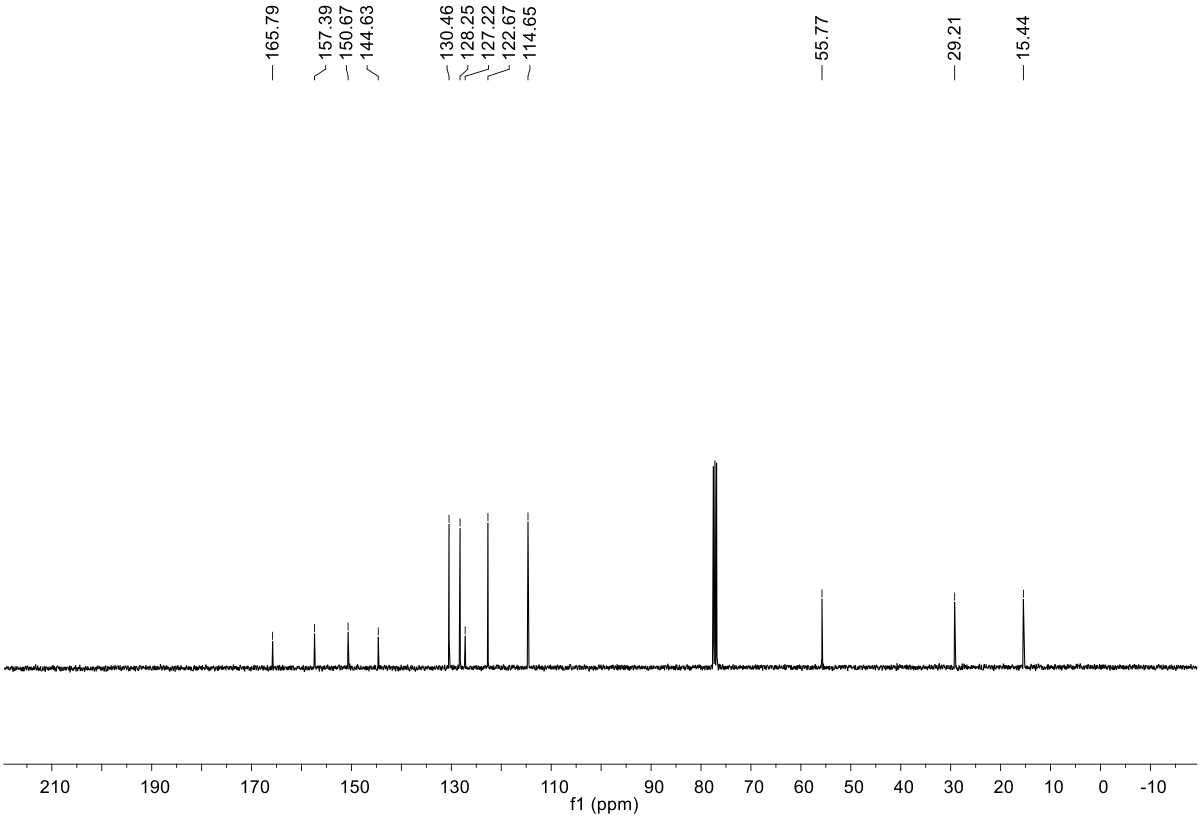
165.79
157.39
150.67
144.63
130.46
128.25
127.22
122.67
114.65
55.77
29.21
15.44
210 190 170 150 130 110 90 80 70 60 50 40 30 20 10 0 -10
f1 (ppm)

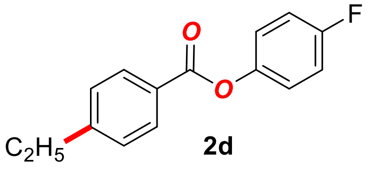
O
O
F
C_2H_5

2d

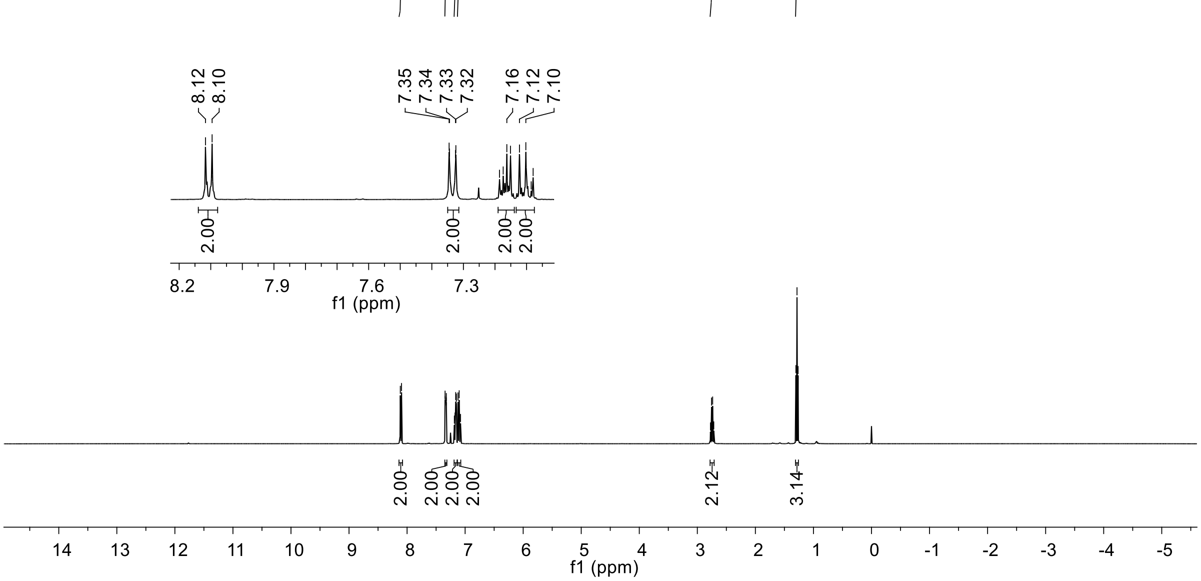
8.12
8.10
7.35
7.34
7.33
7.32
7.19
7.17
7.16
7.15
7.12
7.10
7.09
7.08
2.77
2.76
2.74
2.72
1.30
1.28
1.26
8.12
8.10
7.35
7.34
7.33
7.32
7.16
7.12
7.10
2.00
2.00
2.00
2.00
8.2
7.9
7.6
7.3
f1 (ppm)
2.00
2.00
2.00
2.00
2.12
3.14
14
13
12
11
10
9
8
7
6
5
4
3
2
1
0
-1
-2
-3
-4
-5
f1 (ppm)

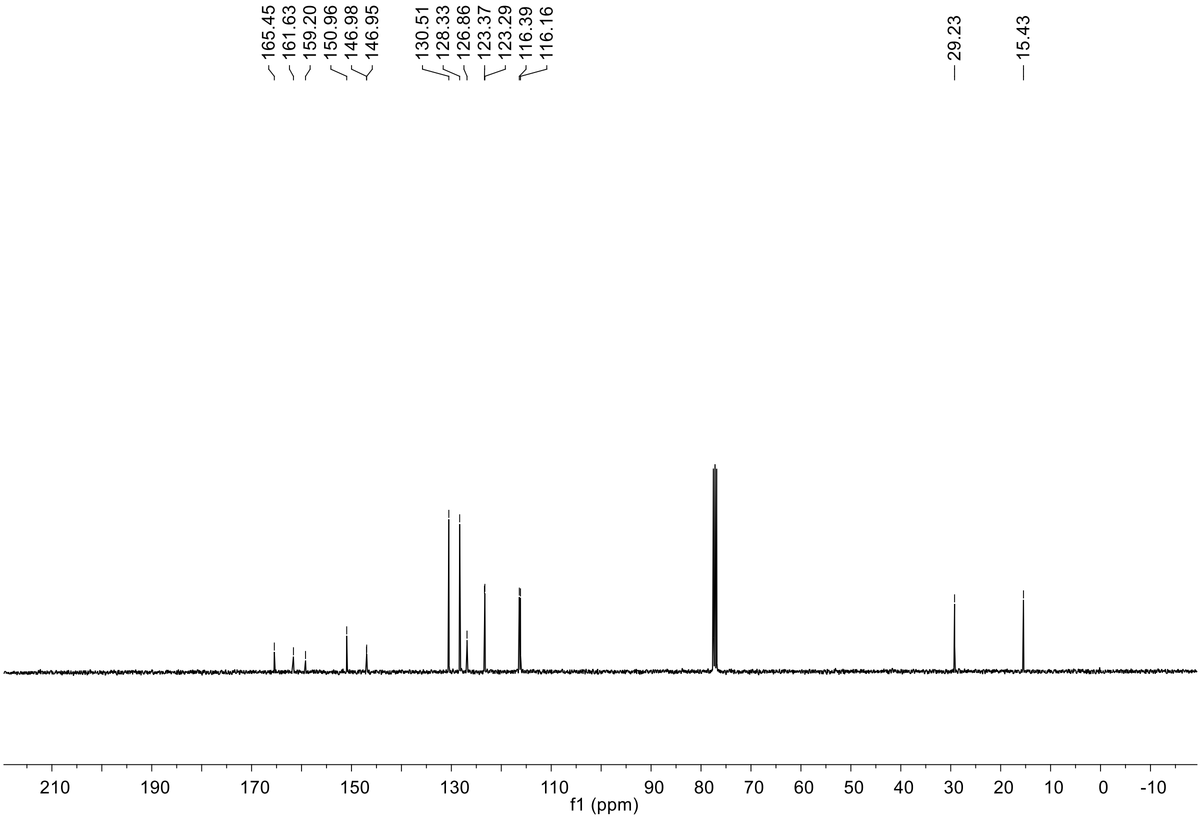
165.45
161.63
159.20
150.96
146.98
146.95
130.51
128.33
126.86
123.37
123.29
116.39
116.16
29.23
15.43
210
190
170
150
130
110
90
80
70
60
50
40
30
20
10
0
-10
f1 (ppm)

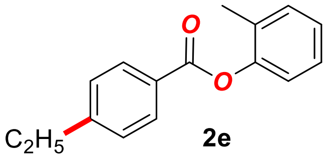
O
O
C_2H_5

2e

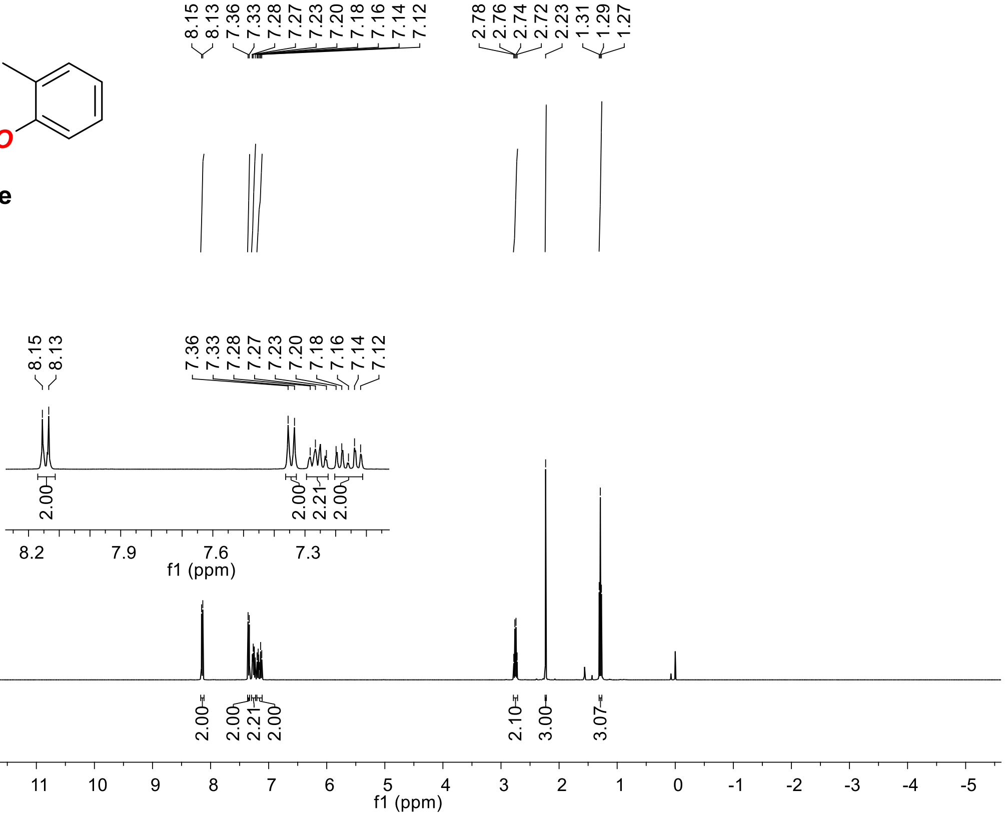
8.15
8.13
7.36
7.33
7.28
7.27
7.23
7.20
7.18
7.16
7.14
7.12
2.78
2.76
2.74
2.72
2.23
1.31
1.29
1.27
2.00
2.00
2.21
2.00
8.2
7.9
7.6
7.3
f1 (ppm)
2.00
2.00
2.21
2.00
2.10
3.00
3.07
14
13
12
11
10
9
8
7
6
5
4
3
2
1
0
-1
-2
-3
-4
-5
f1 (ppm)

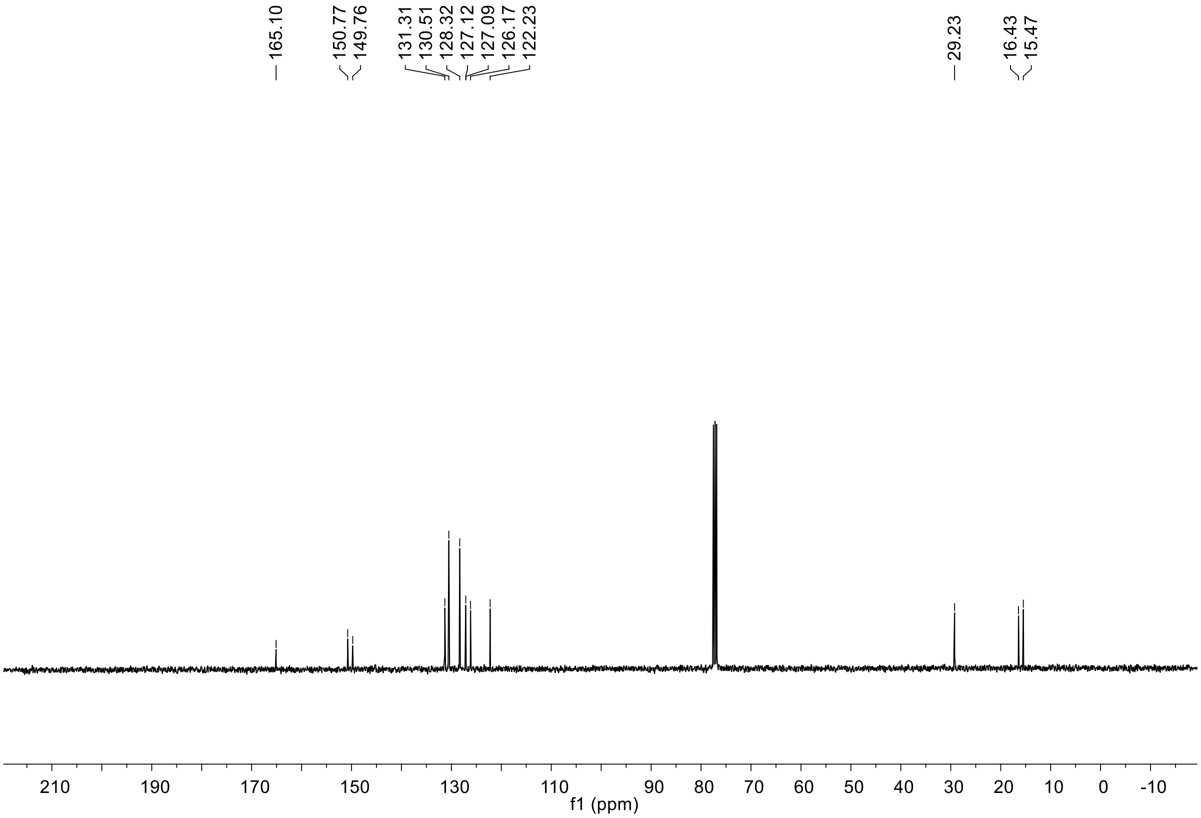
165.10
150.77
149.76
131.31
130.51
128.32
127.12
127.09
126.17
122.23
29.23
16.43
15.47
210
190
170
150
130
110
90
80
70
60
50
40
30
20
10
0
-10
f1 (ppm)

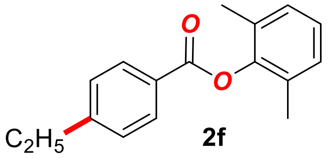
O
O
C_2H_5

2f

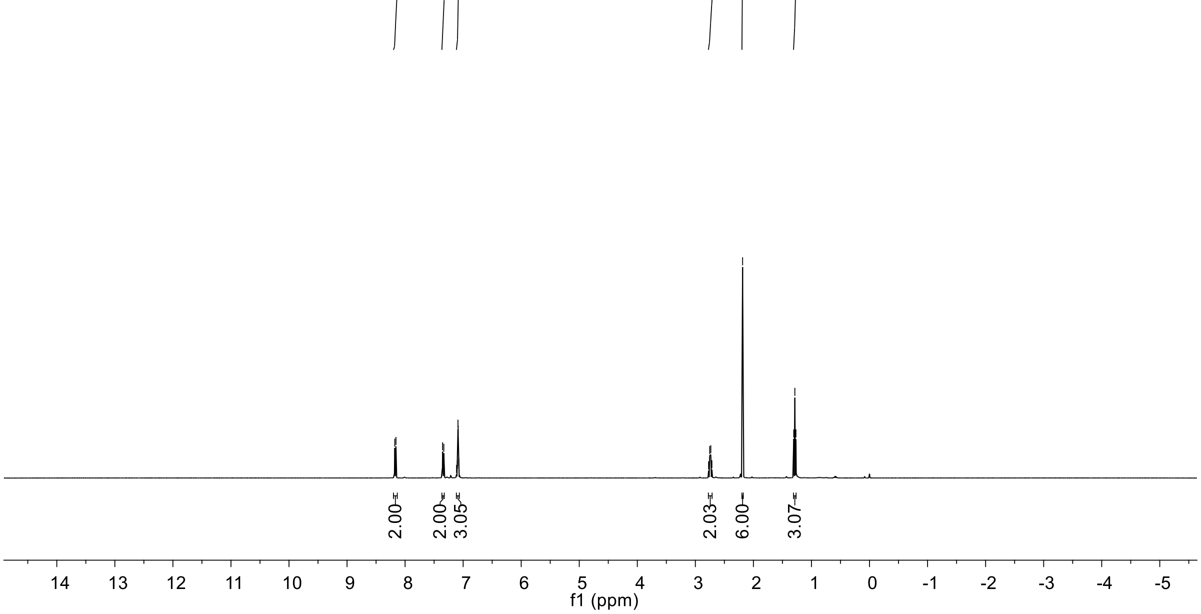
8.18
8.16
7.35
7.33
7.11
7.10
7.09
7.09
7.08
2.77
2.75
2.73
2.71
2.19
1.31
1.29
1.27
2.00
2.00
3.05
2.03
6.00
3.07
14 13 12 11 10 9 8 7 6 5 4 3 2 1 0 -1 -2 -3 -4 -5
f1 (ppm)

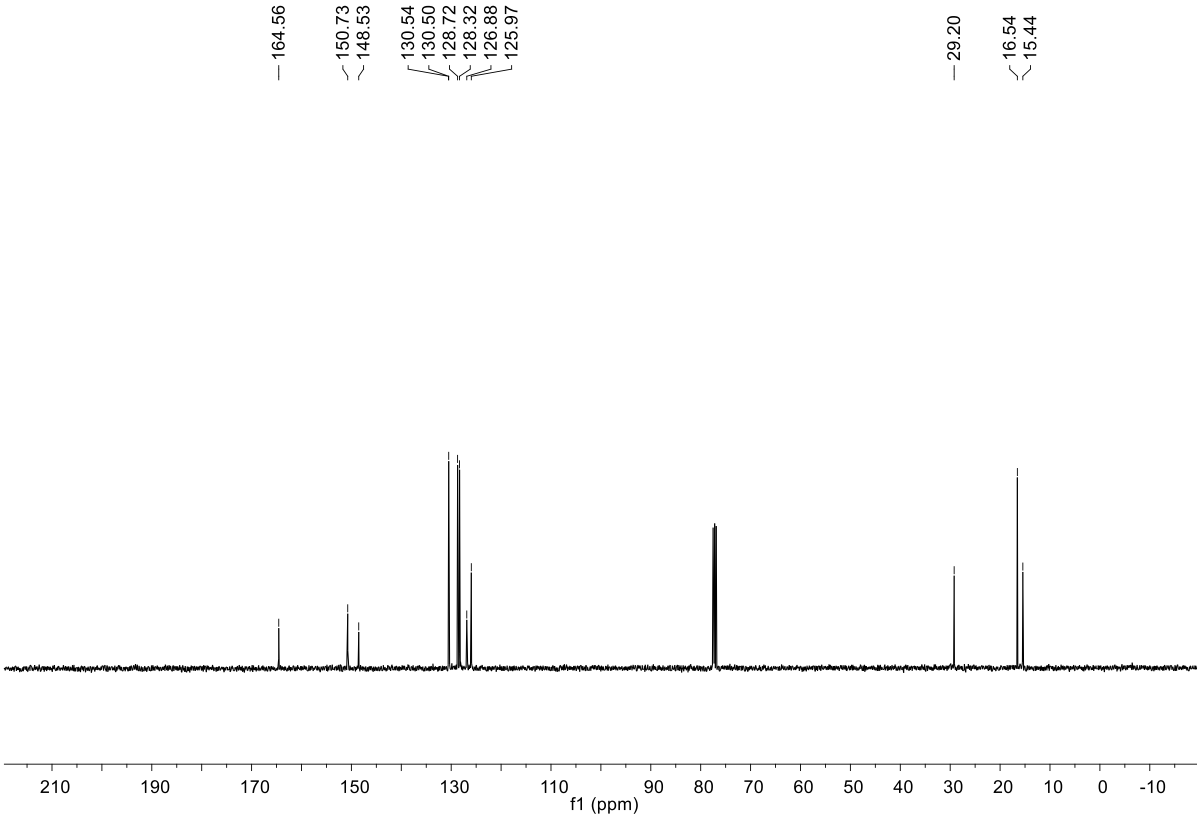
164.56
150.73
148.53
130.54
130.50
128.72
128.32
126.88
125.97
29.20
16.54
15.44
210 190 170 150 130 110 90 80 70 60 50 40 30 20 10 0 -10
f1 (ppm)

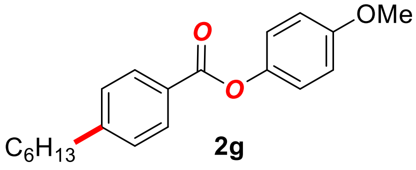
OMe
O
O
C_6H_{13}

2g

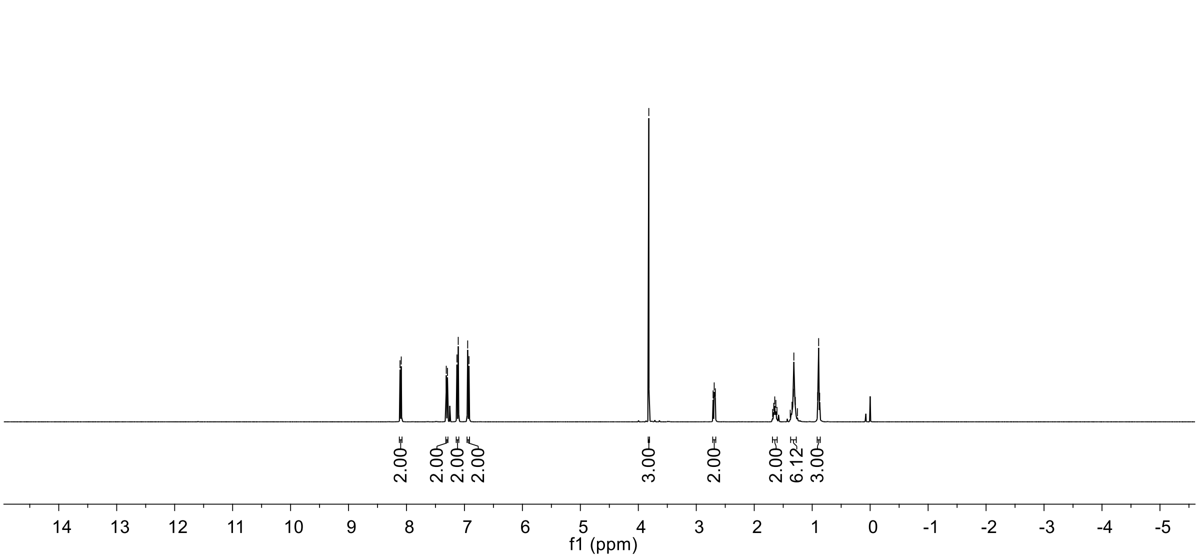
8.11
8.09
7.31
7.29
7.13
7.11
6.94
6.92
3.82
2.71
2.69
2.67
1.68
1.66
1.65
1.63
1.61
1.38
1.35
1.32
1.32
1.29
1.26
0.91
0.89
0.87
2.00
2.00
2.00
2.00
3.00
2.00
2.00
6.12
3.00
14 13 12 11 10 9 8 7 6 5 4 3 2 1 0 -1 -2 -3 -4 -5
f1 (ppm)

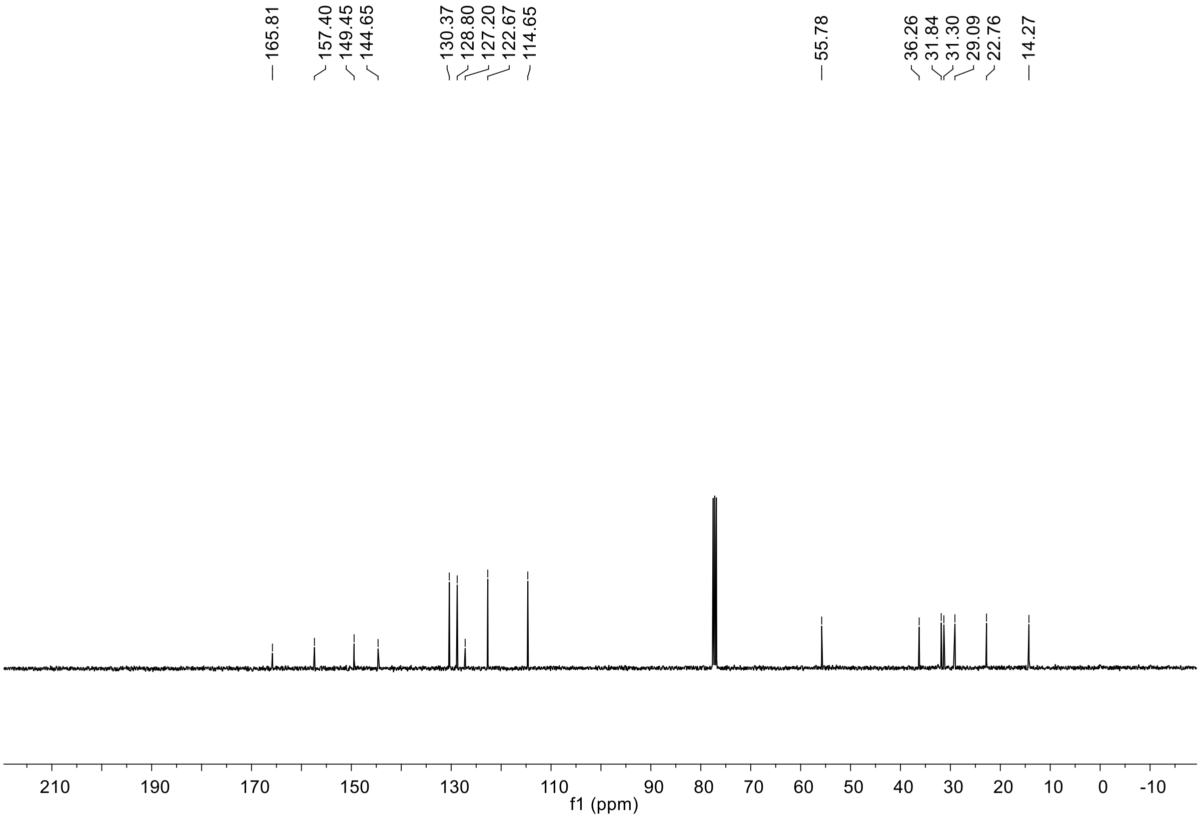
165.81
157.40
149.45
144.65
130.37
128.80
127.20
122.67
114.65
55.78
36.26
31.84
31.30
29.09
22.76
14.27
210 190 170 150 130 110 90 80 70 60 50 40 30 20 10 0 -10
f1 (ppm)

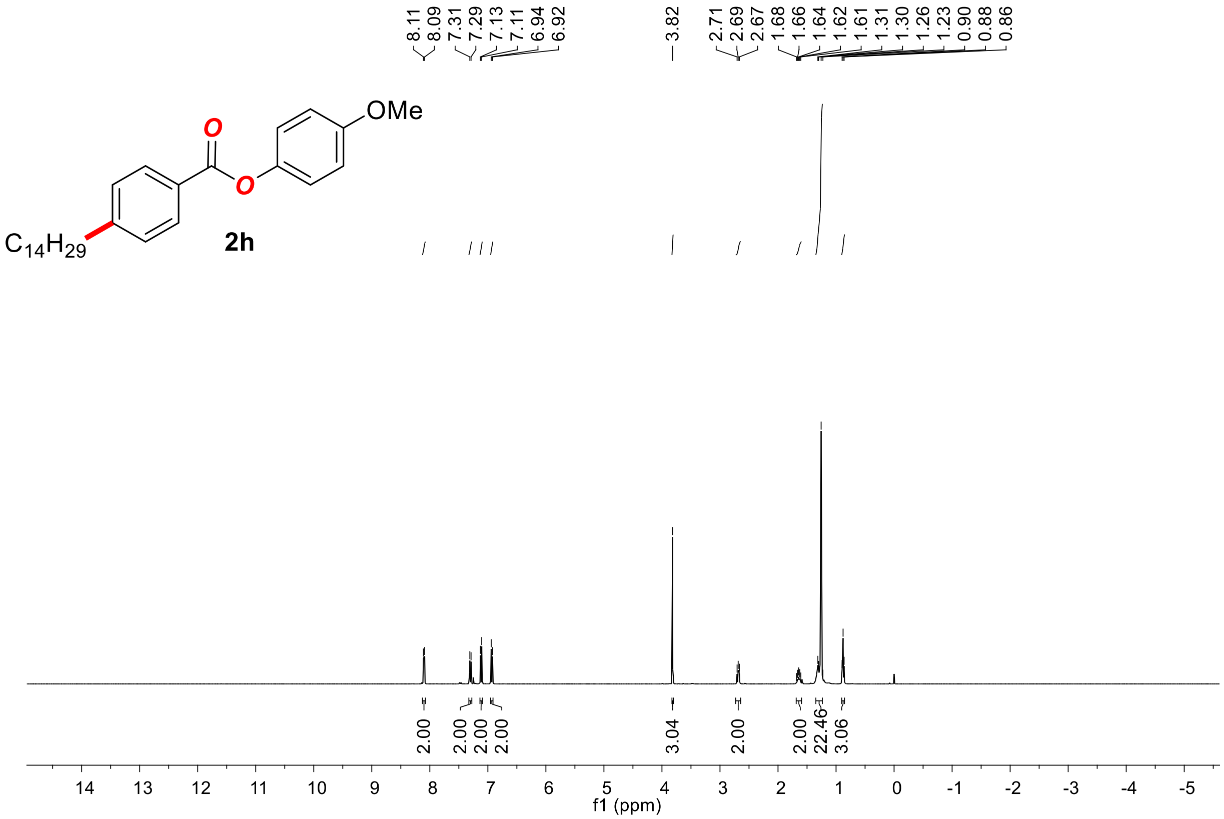
8.11
8.09
7.31
7.29
7.13
7.11
6.94
6.92
3.82
2.71
2.69
2.67
1.68
1.66
1.64
1.62
1.61
1.31
1.30
1.26
1.23
0.90
0.88
0.86
OMe
O
O
C14H29
2.00
2.00
2.00
2.00
3.04
2.00
2.00
22.46
3.06
f1 (ppm)

2h

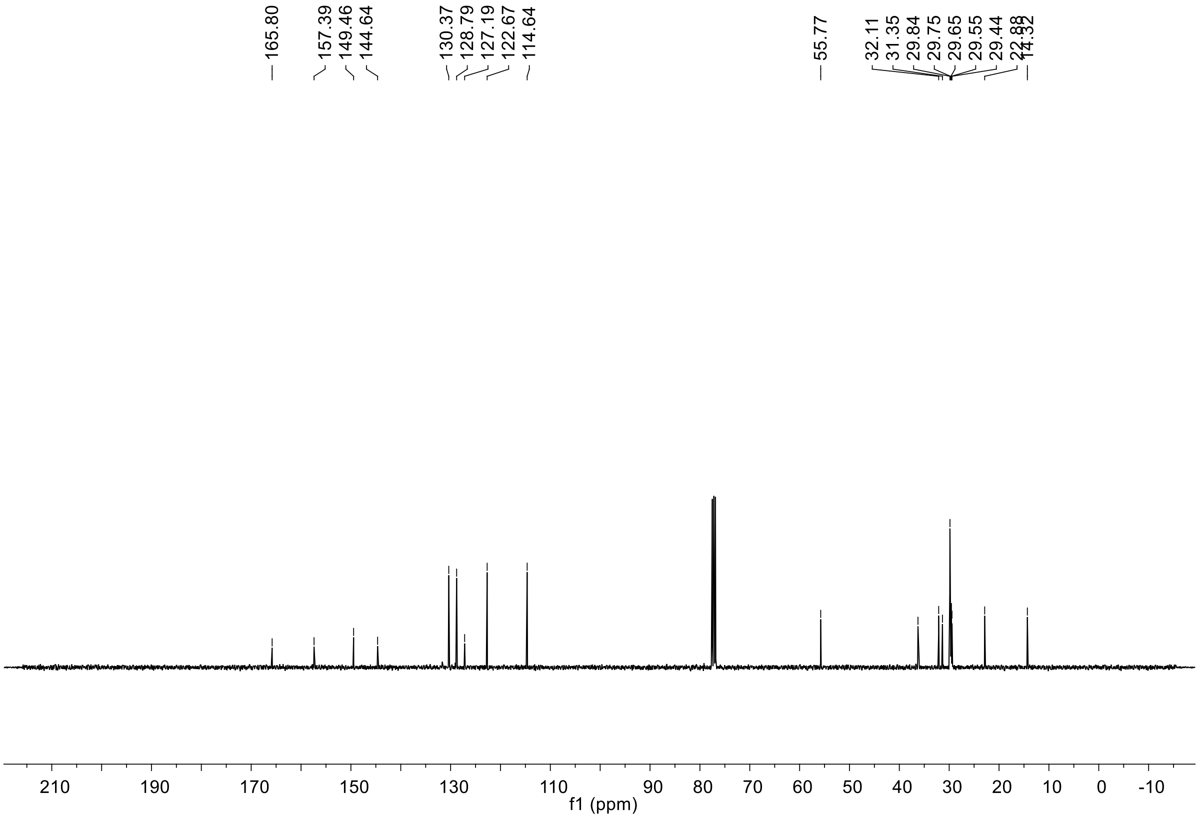
165.80
157.39
149.46
144.64
130.37
128.79
127.19
122.67
114.64
55.77
32.11
31.35
29.84
29.75
29.65
29.55
29.44
22.88
14.32
f1 (ppm)

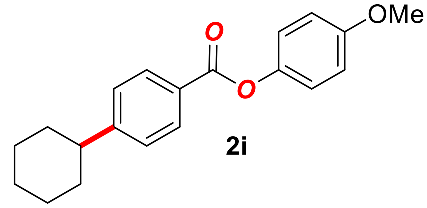
OMe
O
O

2i

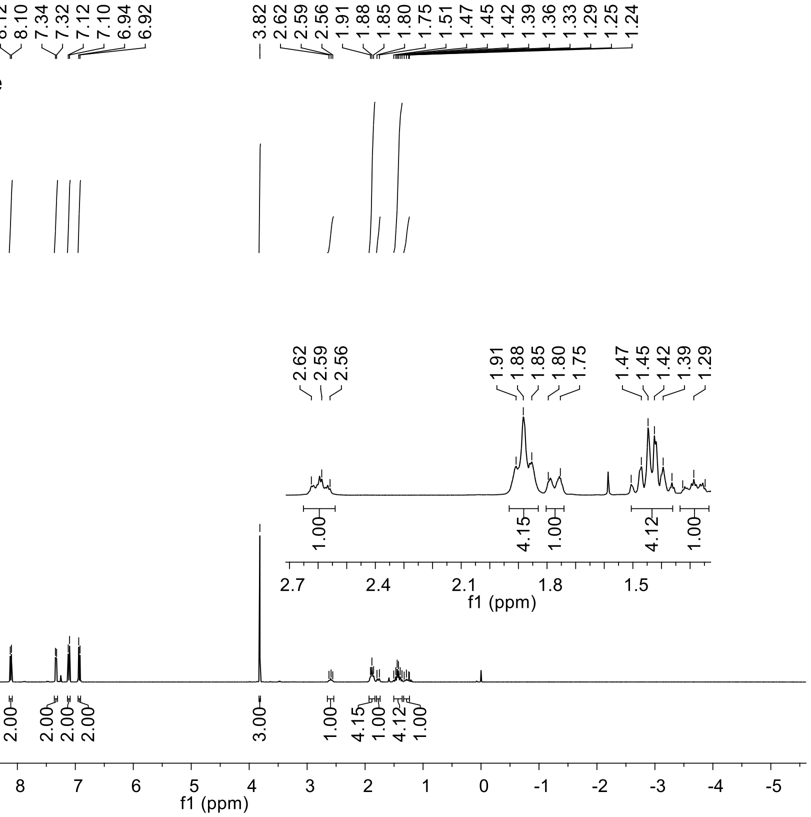
8.12
8.10
7.34
7.32
7.12
7.10
6.94
6.92
3.82
2.62
2.59
2.56
1.91
1.88
1.85
1.80
1.75
1.51
1.47
1.45
1.42
1.39
1.36
1.33
1.29
1.25
1.24
2.00
2.00
2.00
2.00
3.00
1.00
4.15
1.00
4.12
1.00
f1 (ppm)

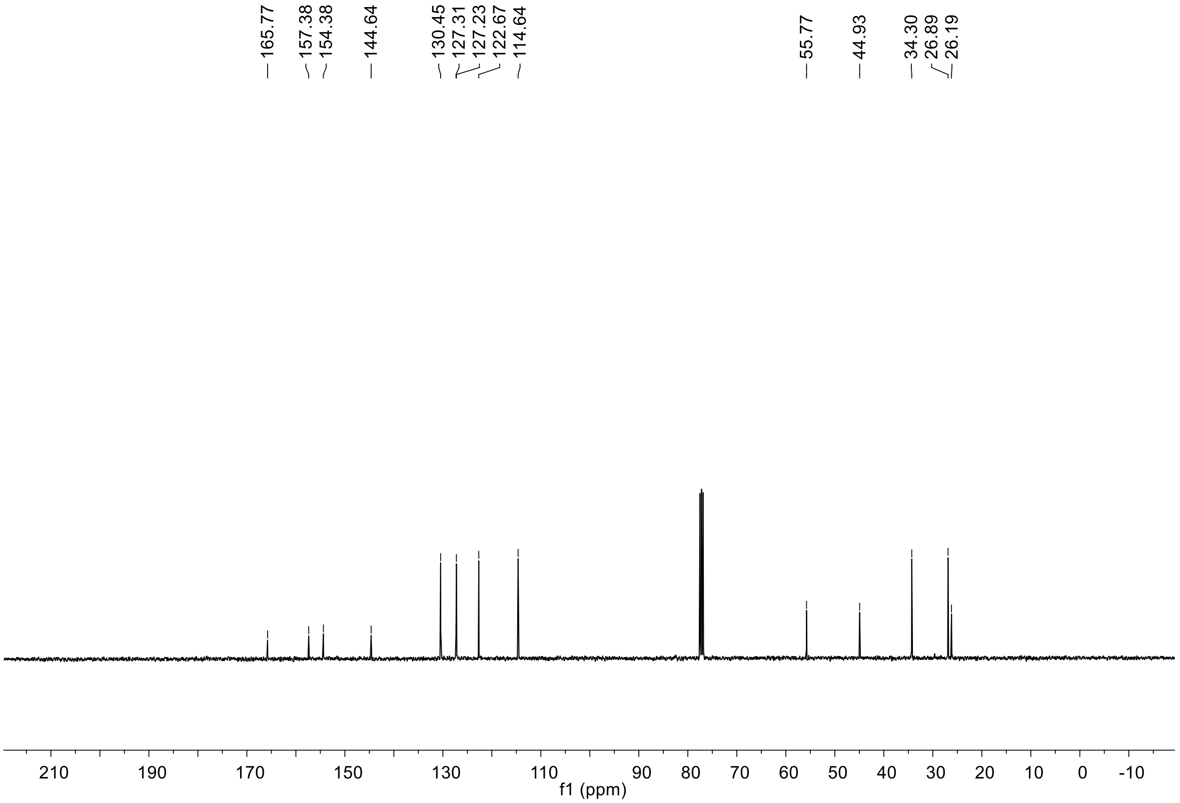
165.77
157.38
154.38
144.64
130.45
127.31
127.23
122.67
114.64
55.77
44.93
34.30
26.89
26.19
f1 (ppm)

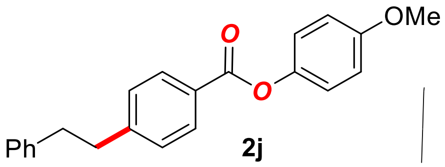
O
O
OMe
Ph

2j

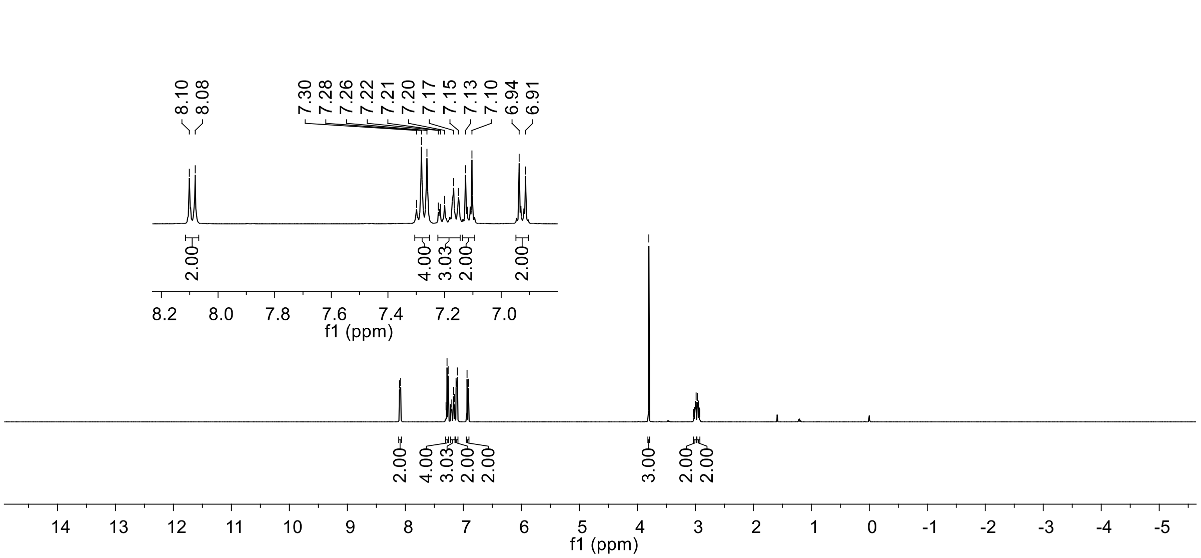
8.10
8.08
7.30
7.28
7.26
7.20
7.17
7.15
7.13
7.10
6.94
6.91
3.80
3.03
3.01
3.00
2.99
2.97
2.95
2.93
2.92
8.10
8.08
7.30
7.28
7.26
7.22
7.21
7.20
7.17
7.15
7.13
7.10
6.94
6.91
2.00
4.00
3.03
2.00
2.00
8.2 8.0 7.8 7.6 7.4 7.2 7.0
f1 (ppm)
2.00
4.00
3.03
2.00
2.00
3.00
2.00
2.00
14 13 12 11 10 9 8 7 6 5 4 3 2 1 0 -1 -2 -3 -4 -5
f1 (ppm)

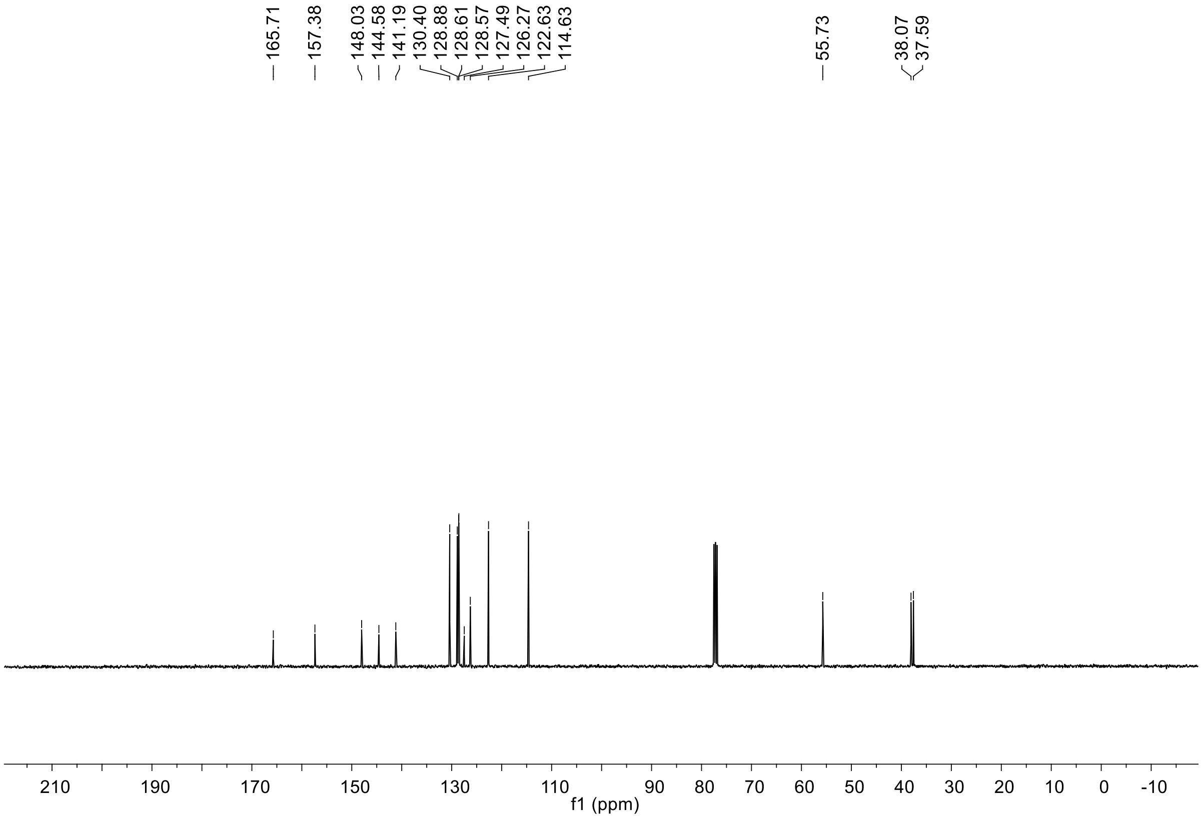
165.71
157.38
148.03
144.58
141.19
130.40
128.88
128.61
128.57
127.49
126.27
122.63
114.63
55.73
38.07
37.59
210 190 170 150 130 110 90 80 70 60 50 40 30 20 10 0 -10
f1 (ppm)
